# Integrated Computational Model of Lung Tissue Bioenergetics

**DOI:** 10.3389/fphys.2019.00191

**Published:** 2019-03-08

**Authors:** Xiao Zhang, Ranjan K. Dash, Anne V. Clough, Dexuan Xie, Elizabeth R. Jacobs, Said H. Audi

**Affiliations:** ^1^Department of Biomedical Engineering, Marquette University, Milwaukee, WI, United States; ^2^Department of Biomedical Engineering, Medical College of Wisconsin, Milwaukee, WI, United States; ^3^Department of Physiology, Medical College of Wisconsin, Milwaukee, WI, United States; ^4^Zablocki V. A. Medical Center, Milwaukee, WI, United States; ^5^Department of Mathematics, Statistics, and Computer Science, Marquette University, Milwaukee, WI, United States; ^6^Department of Mathematical Sciences, University of Wisconsin-Milwaukee, Milwaukee, WI, United States; ^7^Division of Pulmonary and Critical Care Medicine, Medical College of Wisconsin, Milwaukee, WI, United States

**Keywords:** thermodynamically-constrained modeling, cellular metabolism, glycolysis, mitochondrial bioenergetics, isolated rat lungs

## Abstract

Altered lung tissue bioenergetics plays a key role in the pathogenesis of lung diseases. A wealth of information exists regarding the bioenergetic processes in mitochondria isolated from rat lungs, cultured pulmonary endothelial cells, and intact rat lungs under physiological and pathophysiological conditions. However, the interdependence of those processes makes it difficult to quantify the impact of a change in a single or multiple process(es) on overall lung tissue bioenergetics. Integrated computational modeling provides a mechanistic and quantitative framework for the bioenergetic data at different levels of biological organization. The objective of this study was to develop and validate an integrated computational model of lung bioenergetics using existing experimental data from isolated perfused rat lungs. The model expands our recently developed computational model of the bioenergetics of mitochondria isolated from rat lungs by accounting for glucose uptake and phosphorylation, glycolysis, and the pentose phosphate pathway. For the mitochondrial region of the model, values of kinetic parameters were fixed at those estimated in our recent model of the bioenergetics of mitochondria isolated from rat lungs. For the cytosolic region of the model, intrinsic parameters such as apparent Michaelis constants were determined based on previously published enzyme kinetics data, whereas extrinsic parameters such as maximal reaction and transport velocities were estimated by fitting the model solution to published data from isolated rat lungs. The model was then validated by assessing its ability to predict existing experimental data not used for parameter estimation, including relationships between lung nucleotides content, lung lactate production rate, and lung energy charge under different experimental conditions. In addition, the model was used to gain novel insights on how lung tissue glycolytic rate is regulated by exogenous substrates such as glucose and lactate, and assess differences in the bioenergetics of mitochondria isolated from lung tissue and those of mitochondria in intact lungs. To the best of our knowledge, this is the first model of lung tissue bioenergetics. The model provides a mechanistic and quantitative framework for integrating available lung tissue bioenergetics data, and for testing novel hypotheses regarding the role of different cytosolic and mitochondrial processes in lung tissue bioenergetics.

## Introduction

Altered lung tissue bioenergetics (i.e., cellular capacity for ATP production) is an important early step in the pathogenesis of lung diseases (Bongard et al., 2013; Kallet and Matthay, 2013), including acute lung injury (ALI), which is one the most frequent causes of admission to medical intensive care units (Kallet and Matthay, 2013). A wealth of information exists regarding the bioenergetic processes in mitochondria isolated from rat lungs, cultured pulmonary endothelial cells, and intact rat lungs under physiological and pathophysiological conditions (Fisher et al., 1976; Kerr et al., 1979; Fisher and Dodia, 1981, 1984; Fisher, 1984; Kallet and Matthay, 2013; Zhang et al., 2018). However, the interdependence of lung cellular processes makes it difficult to quantify the impact of a change in a single or multiple cellular process(es) on overall lung tissue bioenergetics. Furthermore, it is difficult to integrate bioenergetic data measured at different levels of cellular organization. For instance, although ~85% of cellular ATP is produced in mitochondria under physiological conditions (Fisher, 1984), glycolysis is important for lung tissue bioenergetics since it can partially compensate for the decrease in lung tissue ATP when mitochondrial ATP generation is impaired (Tierney and Young, 2011).

**Figure 1:**
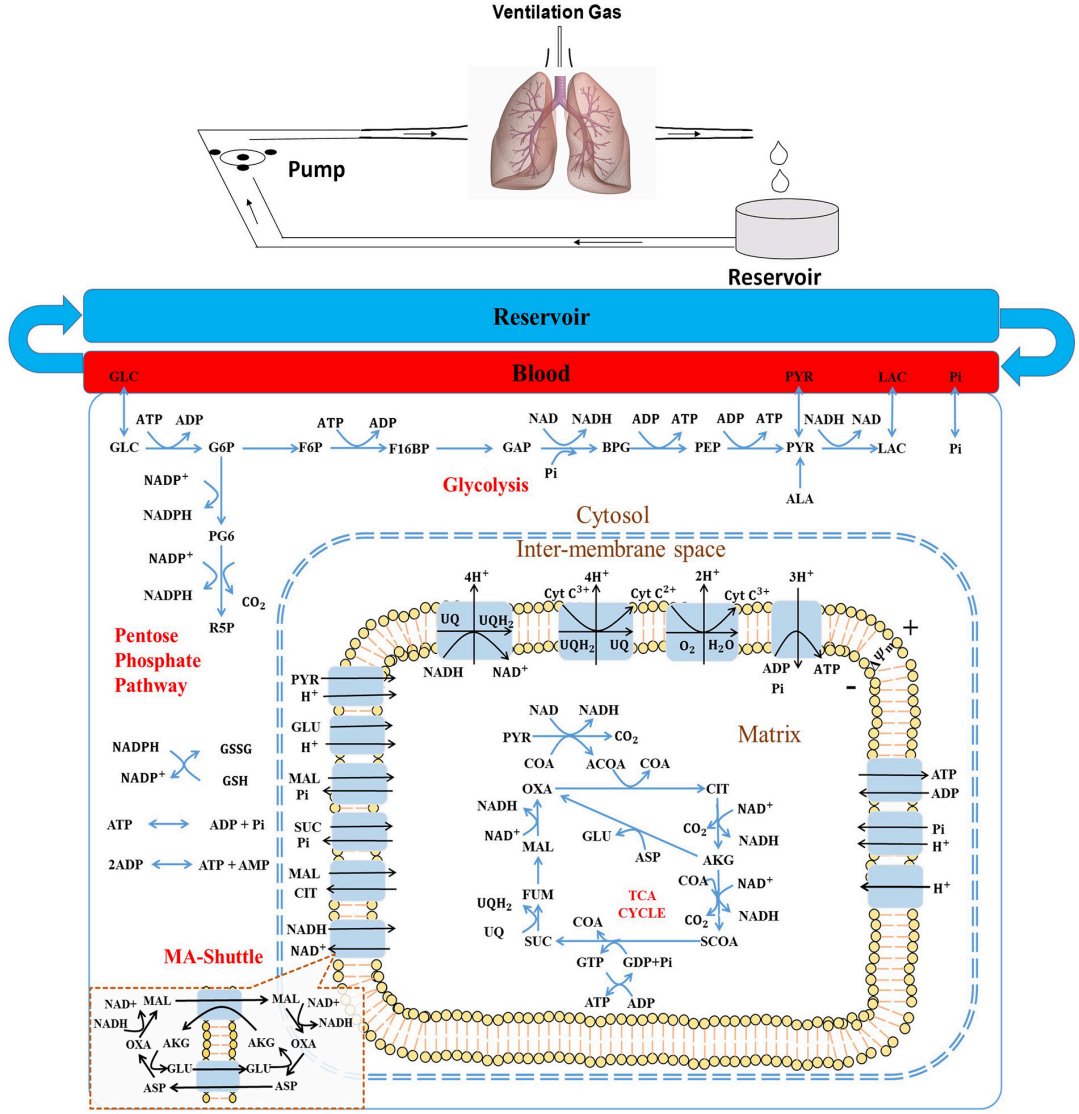
**(Top)** Schematic of the isolated rat lung ventilation-perfusion system. **(Bottom)** Structure of the rat lung tissue bioenergetics model, which consists of five regions: The reservoir + tubing region, the lung vascular (blood) region, the cytosolic region, the inter-membrane space (IMS) region, and the mitochondria matrix region. Major reactions include glycolysis, pentose phosphate cycle, TCA cycle, and electron transport chain reactions. Major biochemical species include: GLC, glucose; G6P, glucose 6-phosphate; F6P, fructose 6-phospate; F16BP, Fructose 1,6-biphosphate; GAP, Glyceraldehyde 3-phosphate; BPG, 1,3-Bisposphoglycerate; PEP, Phosphoenolpyruvate; PYR, pyruvate; LAC, lactate; ALA, alanine; CoA, coenzyme-A; ACoA, acetyl-CoA; OXA, oxaloacetate; CIT, citrate; AKG, a-ketogluterate; SCoA, succinyl-CoA; SUC, succinate; FUM, fumarate; MAL, malate, GLU, glutamate; and ASP, aspartate; NAD and NADH, oxidized and reduced form of nicotinamide adenine dinucleotide, respectively; ADP and ATP, adenosine triphosphate and adenosine diphosphate, respectively; UQ and UQH_2_, oxidized and reduced form of ubiquinone; respectively, CtyC^3+^ and CytC^2+^, oxidized and reduced form of cytochrome c, respectively. MA-shuttle, malate-aspartate shuttle (insert).

Integrating bioenergetics data from isolated mitochondria, cultured cells, and the whole-organ is necessary for determining the functional significance of targeting a specific cellular process for prognostic and/or therapeutic purposes. Integrated computational modeling provides a mechanistic and quantitative framework for doing that. Recently, we developed and validated a thermodynamically-constrained integrated computational model of the bioenergetics of isolated lung mitochondria (Zhang et al., 2018). Simulations using that model provided important insights into the bioenergetics and respiration of mitochondria isolated from lung tissue and how they differ from those of mitochondria isolated from other organs (Wu et al., 2007; Bazil et al., 2010). The isolated perfused rat lung preparation (Figure 1, top panel) allows us to control the composition of lung perfusate and ventilation gas, and to directly manipulate specific key cellular pathways pertinent to lung tissue bioenergetics (Fisher et al., 1976; Kerr et al., 1979; Fisher and Dodia, 1981, 1984; Fisher, 1984). Previous studies carried out using an isolated perfused rat lung preparation provide a wealth of information regarding lung tissue bioenergetics, such as glucose uptake, lactate, and pyruvate production rate, ATP content, surface NADH, oximetry, etc. (Bassett and Fisher, 1976b; Fisher and Dodia, 1981; Fisher, 1984; Sepehr et al., 2013). Again, the interdependence among cytosolic and mitochondrial processes is not clear from those data, including how a change in one or more cytosolic or mitochondrial processes alters overall lung tissue bioenergetics. Moreover, it is difficult to integrate mitochondrial data from isolated lung mitochondria with data from intact lungs or lung tissue homogenate.

The objective of this study is to develop and validate an integrated computational model of intact rat lung tissue bioenergetics using existing experimental data. The model expands our recently developed integrated computational model of the bioenergetics of isolated lung mitochondria by accounting for glucose uptake and phosphorylation, glycolysis, and the pentose phosphate pathway. To the best of our knowledge, this is the first model for intact lung tissue bioenergetics. The model provides important insights into how different cellular pathways, such as glycolysis, are regulated by different substrates. In addition, the model provides a mechanistic and quantitative framework for integrating available lung tissue bioenergetic data, and for testing novel hypotheses regarding the role of different cytosolic and mitochondrial processes in lung tissue bioenergetics under physiological and pathophysiological conditions.

## Methods

### Model Development

Our recently developed and validated integrated computational model of the bioenergetics of mitochondria isolated from rat lungs forms the foundation of the integrated computational model of rat lung tissue bioenergetics (Zhang et al., 2018). The model consists of five different regions (Figure 1, bottom panel): The reservoir + tubing region, the lung vascular (blood) region, the cytosolic region, inter-membrane space (IMS) region, and the mitochondria matrix region. For all regions, the general forms of the reaction and transport fluxes are identical to those developed for the integrated computational model of the bioenergetics of mitochondria isolated from rat lungs (Zhang et al., 2018) (see Supporting Information).

Lung tissue is capable of oxidizing a wide range of substrates, including glucose, fatty acids, amino acids, and lactate (Kerr et al., 1979; Fisher, 1984; Fisher and Dodia, 1984). However, glucose is by far the major substrate under physiological conditions (Fisher, 1984). Alanine, an amino acid, can also be an important substrate, especially when glucose is low (Kerr et al., 1979; Kadlecek et al., 2014). Thus, glucose and alanine utilization and intermediary metabolism are also accounted for. The model does not account for glycogen, which is low and relatively constant in lung tissue (Kerr et al., 1979).

The model accounts for 70 state variables, including the concentrations of key metabolites in the mitochondrial matrix region and cytosol, as well as the mitochondrial membrane potential. Volumes of each region and general model parameters are listed in Table 1. Key reactions in the glycolysis pathway and pentose phosphate pathway are also included (Figure 1, bottom panel).

**Table 1:** General model parameter values.

**Parameter**	**Value (units)**	**References**
**VOLUMES**		
Capillary vascular volume (V_b_)	0.66 (ml)	Crapo et al., 1980
Total tissue volume (V_c_+V_m_+V_i_)	0.67 (ml)	Crapo et al., 1980
Total lung volume	1.33 (ml)	Crapo et al., 1980
Lung tissue cytosolic volume (V_c_)	0.66 (ml)	Gan et al., 2011*
Mitochondria volume (V_m_)	13.12 (μl)	Gan et al., 2011*
Inter-membrane space volume (V_i_)	0.95 (μl)	Wu et al., 2007*
Lung wet/dry weight ratio	5.87 ± 0.24	Bongard et al., 2013
Average rat lung dry weight	0.227 (g)	Bongard et al., 2013**
**METABOLIC POOLS**		
Total pyridine nucleotide content (NAD^+^ + NADH) in mitochondria	1.73 (nmol/mg mitochondria)	Fisher et al., 1973
Cytosolic NAD^+^ concentration	340 (μM)	Kadlecek et al., 2014

* *Calculated based on V_m_/V_c_ = 1/50 (Gan et al., 2011), and V_i_/V_m_ = 0.0724 (Wu et al., 2007). Values are mean ± SE*.

** *Calculated from lung wet weight based on rat lung wet weight/ dry weight ratio = 5.87*.

Thirteen trans-membrane transport processes are included in the model to account for the exchange of key metabolic species between the mitochondrial matrix and cytosol, and between the blood region and cytosolic region. We used reaction and transport flux expressions similar to those in our model of the bioenergetics of isolated rat lung mitochondria (Zhang et al., 2018). For instance, for the following general multi-substrate and multi-product enzymatic reaction

(1) $$\begin{array}{l} \overset{N_{s}}{\underset{i=1}{\sum }}\alpha_{i}S_{i}↔\overset{N_{P}}{\underset{j=1}{\sum }}\beta_{j}P_{j}, \end{array}$$

the general form of the reaction flux, *J*, accounting for the thermodynamic (Haldane) constraint, is given by:

(2) $$\begin{array}{l} J=\frac{\frac{V_{maxf}}{\prod_{i=1}^{N_{s}}K_{S_{i}}^{\alpha_{i}}}\left(\prod_{i=1}^{N_{s}}{\left[S_{i}\right]}^{\alpha_{i}}-\frac{\prod_{j=1}^{N_{p}}{\left[P_{j}\right]}^{\beta_{j}}}{{K^{\prime }}_{eq}}\right)}{\prod_{i=1}^{N_{s}}\left(1+\frac{{\left[S_{i}\right]}^{\alpha_{i}}}{K_{S_{i}}^{\alpha_{i}}}\right)\times \prod_{j=1}^{N_{p}}\left(1+\frac{{\left[P_{j}\right]}^{\beta_{j}}}{K_{P_{j}}^{\beta_{j}}}\right)} \end{array}$$

where *S*_*i*_ is the i^th^ substrate, *P*_*j*_ is the j^th^ product, *N*_*s*_ and *N*_*p*_ are the number of substrates and products, respectively, α_*i*_ and β_*j*_ are the corresponding stoichiometric coefficients, *K*_*Si*_ and *K*_*Pj*_ are the apparent Michaelis constants corresponding to substrates and products, respectively; [*S*_*i*_]and[*P*_*j*_] are the concentrations of substrate *i* and product *j*, respectively; *V*_*maxf*_ is the maximum forward reaction rate; and *K*${}_{eq}^{{}^{\prime }}$ is the apparent equilibrium constant for the reaction, which is the value of the equilibrium-state reaction quotient (i.e., ratio of the product of product concentrations over the product of substrate concentrations) at specified thermodynamic conditions (i.e., temperature, ionic strength, and pH).

The lung bioenergetics model accounts for the pH dependence of the apparent equilibrium constants for proton-releasing reactions (Equation 3a) and for proton-consumption reactions (Equation 3b) (Alberty, 1998; Dash and Beard, 2008):(Bazil et al., 2010):

(3a) $$\begin{array}{l} {K^{\prime }}_{eq}=K_{eq}^{\prime 0}\times 10^{pH-7}=e^{-\Delta_{r}G^{\prime 0}/RT}\times 10^{pH-7} \end{array}$$

(3b) $$\begin{array}{l} {K^{\prime }}_{eq}=K_{eq}^{\prime 0}\times 10^{7-pH}=e^{-\Delta_{r}G^{\prime 0}/RT}\times 10^{7-pH} \end{array}$$

where $K_{\text{eq}}^{\prime 0}$ is the reaction's apparent equilibrium constant (${K^{\prime }}_{eq}$) at pH of 7, and $\Delta_{\text{r}}{G{}^{\prime }}^{0}$ *R* and *T* are the standard Gibbs free energy of the reaction at pH = 7, gas constant, and temperature, respectively. In the presence of cofactor pairs (e.g., NADH and NAD^+^, ATP and ADP, GTP and GDP, CoA and ACoA, or CoA and SCoA), the generalized reaction flux Equation 2 was modified appropriately so as not to include any interactive cofactor product terms (Wagner, 1976; Zhang et al., 2018). The assumption is that the substrate and product represented as a cofactor pair, bind with a given enzyme at the same binding site, so that the resulting reaction flux expression does not include the corresponding substrate and product multiplication term in the denominator of Equation 2 (Zhang et al., 2018). Under such conditions, the form of Equation 2 for a two cofactor pairs *S*_1_ and *P*_1_ and *S*_2_ and *P*_2_ becomes Equation 4 instead of Equation 5 [see derivation of Equations 4 and 5 on pages 6-8 of the supplement for (Zhang et al., 2018)].

(4) $$\begin{array}{lll} J & = & \frac{\frac{V_{maxf}}{K_{S1}K_{S2}}\left(\left[S_{1}\right]\left[S_{2}\right]-\frac{\left[P_{1}\right]\left[P_{2}\right]}{{K^{\prime }}_{eq}}\right)}{\left(1+\frac{\left[S_{1}\right]}{K_{S1}}+\frac{\left[P_{1}\right]}{K_{P1}}\right)\left(1+\frac{\left[S_{2}\right]}{K_{S2}}+\frac{\left[P_{2}\right]}{K_{P2}}\right)} \end{array}$$

(5) $$\begin{array}{lll} J & = & \frac{\frac{V_{maxf}}{K_{S1}K_{S2}}\left(\left[S_{1}\right]\left[S_{2}\right]-\frac{\left[P_{1}\right]\left[P_{2}\right]}{{K^{\prime }}_{eq}}\right)}{\left(1+\frac{\left[S_{1}\right]}{K_{S1}}\right)\left(1+\frac{\left[S_{2}\right]}{K_{S2}}\right)\left(1+\frac{\left[P_{1}\right]}{K_{P1}}\right)\left(1+\frac{\left[P_{2}\right]}{K_{P2}}\right)} \end{array}$$

Furthermore, Equation (2) can be suitably modified to account for other reaction kinetic mechanisms (e.g., sequential-ordered, ping-pong) (Zhang et al., 2018). All the reactions and transport processes and the associated flux expressions used in the model are listed in the Supporting Information.

Several glycolytic reactions are regulated by specific activators or inhibitors (Berg et al., 2002). For instance, hexokinase (HK) is inhibited by its reaction product glucose-6-phosphate (G6P), phosphofructokinase (PFK) is known to be activated by cytosolic AMP and inhibited by cytosolic ATP and citrate (Heesbeen et al., 1989; Berg et al., 2002). Those reaction fluxes are modified to account for such regulatory effects as described in the Supporting Information.

### Governing Ordinary Differential Equations for the Lung Tissue Bioenergetics Model

The governing ordinary differential equations (ODEs) describing the dynamic changes in the concentrations (*C*) of various chemical species in different regions (total 70 state variables) were derived based on the principle of mass balance. The change in the concentration of a given species within each of the five regions is given by:

(6a) $$\begin{array}{lll} V_{r}\frac{dC_{r,j}}{dt} & = & F\left(C_{b,j}-C_{r,j}\right) \end{array}$$

(6b) $$\begin{array}{lll} V_{b}\frac{dC_{b,j}}{dt} & = & F\left(C_{r,j}-C_{b,j}\right)-\sum J_{b-c,j} \end{array}$$

(6c) $$\begin{array}{lll} \;V_{c}\frac{dC_{c,j}}{dt} & = & \sum \alpha_{c,j}J_{c,j}+\sum J_{b-c,j}-\sum J_{c-m,j} \end{array}$$

(6d) $$\begin{array}{lll} V_{m}\frac{dC_{m,j}}{dt} & = & \sum \alpha_{m,j}J_{m,j}+\sum J_{c-m,j} \end{array}$$

(6e) $$\begin{array}{lll} V_{i}\frac{dC_{i,j}}{dt} & = & \sum \alpha_{i,j}J_{i,j}-\sum J_{i-m,j} \end{array}$$

where *C*_*x, j*_is the concentration of the j^th^ species in region *x, F* is the flow rate, *J*_*x, j*_ is the j^th^ reaction flux in region *x*, and *J*_*x*−*y, j*_ is the j^th^ transport flux between region *x* and region *y*, and *V*_*x*_ is the volume of region *x*. Subscripts *r, b, c, m*, and *i* denote reservoir region, blood region, cytosolic region, mitochondrial region, and inter-membrane space region, respectively. Detailed mass balance equations are included in the Supporting Information.

The model accounts for both components (electrical gradient and pH gradient) of the proton motive force (Δ*G*_*H*_) that drive several reaction and transport processes in the mitochondria, as established in our recently published article (Zhang et al., 2018) and as described in the Supporting Information. In the model, Δ*G*_*H*_ is defined as:

(7) $$\begin{array}{l} \Delta G_{H}=F\Delta {\text{Ψ}}_{m}+\left(RT\right)ln\left(\left[H_{i}^{+}\right]/\left[H_{m}^{+}\right]\right) \end{array}$$

where ΔΨ_m_ is the mitochondrial membrane potential, and $\left[H_{m}^{+}\right]\;$ and $\left[H_{i}^{+}\right]$ are the proton concentrations in the mitochondrial matrix and inner membrane space, respectively (Wu et al., 2007; Dash and Beard, 2008; Zhang et al., 2018). The rates of change in ΔΨ_m_ and $H_{m}^{+}\;$ are described by equations A111 and A108 in the Supporting Information. The proton concentration in the inner membrane space, [$H_{i}^{+}]$, is assumed to be the same as that in the cytosolic region, which in turn is assumed to be constant because of the high proton buffering capacity of the bicarbonate and phosphate buffering systems in the cytosol (Li et al., 2009). It is worth noting that the above expression for Δ*G*_*H*_ is used for all the reaction and transport fluxes affected by it (e.g., ETC complexes I, III, IV, and IV, see Supporting Information).

The model was implemented in MATLAB (MathWorks Inc., Natrick, MA) and the MATLAB function “*ode15s*” was used to solve the system of governing ODEs (see Supporting Information). This MATLAB solver is appropriate for solving stiff ODEs, which is usually a characteristics of metabolic models due to the presence of a wide range of time-scales for the reactions of the different enzymes and transporters governing the dynamics of metabolites' concentrations within the different regions of the model.

### Estimation of Model Parameters

**Figure 2:**
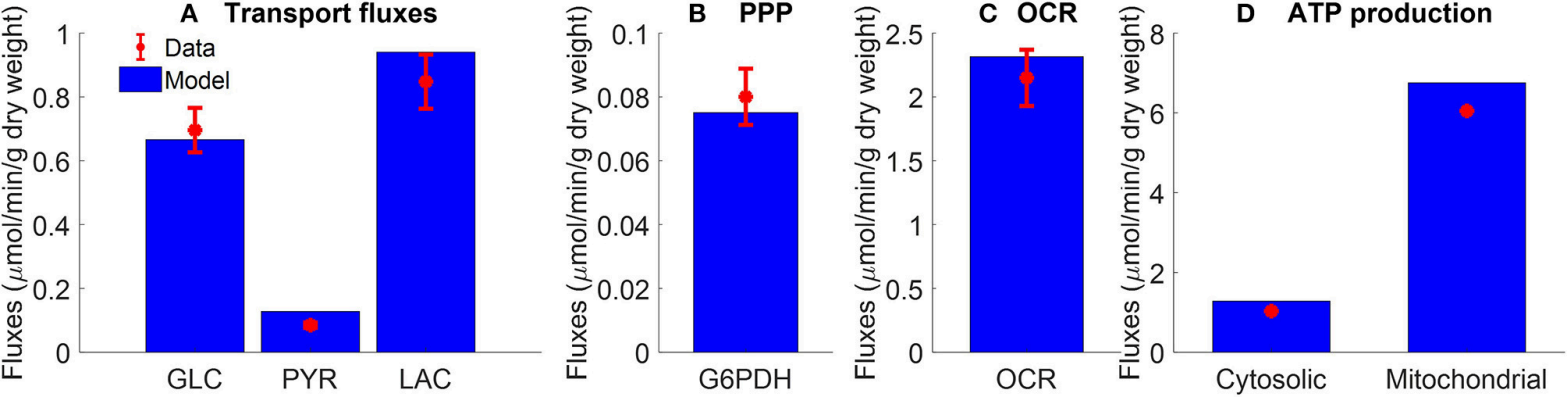
Pseudo-steady state reaction fluxes used for parameter estimation. **(A)** Glucose (GLC) consumption rate (Kerr et al., 1979) and total lactate (LAC) and pyruvate (PYR) production rates (Fisher and Dodia, 1981) of isolated rat lungs perfused with perfusate containing 5.5 mM glucose. Red symbols are experimental data (mean ± SE, *n* = 4). **(B)** Pseudo-steady state reaction flux of pentose phosphate pathway (PPP) measured at the end of 100 min recirculation time (Fisher, 1984). G6PDH: glucose 6 phosphate dehydrogenase. Red symbol is experimental data (mean ± SE, *n* = 4) calculated based on (Bassett and Fisher, 1976b; Fisher, 1984). **(C)** Lung cyanide-sensitive oxygen consumption rate (OCR), calculated as lung OCR in the absence of potassium cyanide—OCR in the presence of potassium cyanide (complex IV inhibitor, 2 mM) of atelectatic isolated perfused rat lungs (Audi et al., 2003). Red symbol is experimental data (mean ± SE, *n* = 6). For the model, lung OCR was calculated as half of reaction flux of complex IV. **(D)** Mitochondrial and cytosolic ATP production rate were estimated based on recovery of catabolic products from glucose (Fisher, 1984). Units are converted from μmol/h/g dry weight to μmol/min/g dry weight. Red symbols are experimental data. In model simulations, pseudo-steady state reaction fluxes are collected at the end of 100 min simulation time. For all four panels, blue bars are model fits to the data.

**Figure 3:**
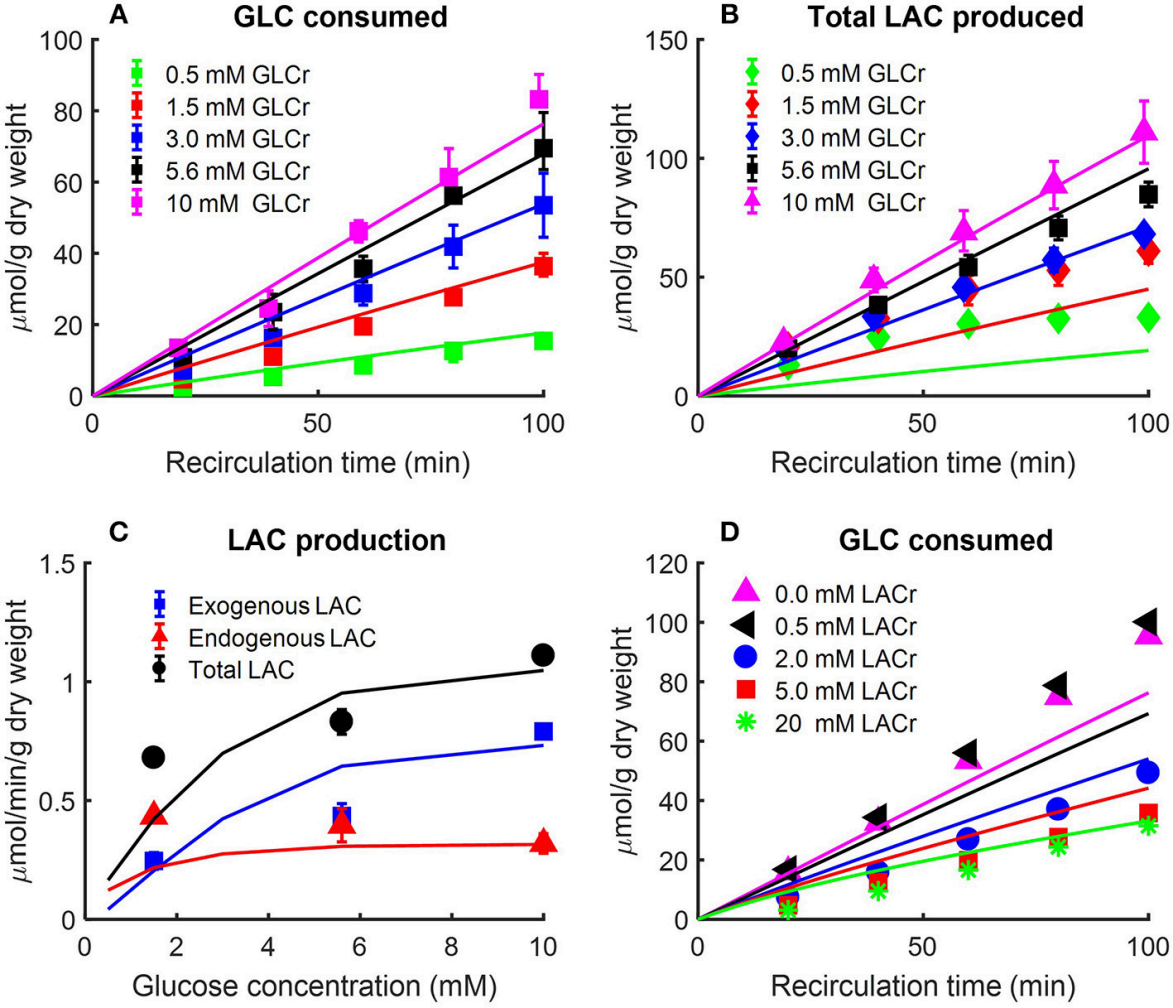
**(A)** Effect of perfusate glucose (GLC) concentration on rat lung glucose consumption rate (Kerr et al., 1979). Lungs were isolated from male Sprague-Dawley rats (200–260 g) and perfused with different concentrations of D-glucose plus 5-^3^H-glucose and U-^14^C glucose. The labeled glucose, 5-^3^H-glucose, and U-^14^C glucose were used to measure glucose consumption and exogenous lactate production, respectively. The production rate of ^3^H_2_O was used as index of glucose consumption since more than 90% of ^3^H from glucose was recovered as ^3^H_2_O (Kerr et al., 1979). Values are mean ± SE (*n* = 3 or more for each glucose concentration). Lines are model fits to data. **(B,C)** Lung exogenous, endogenous, and total lactate (LAC) production rates as a function of perfusate glucose concentration (Kerr et al., 1979). Isolated rat lungs were perfused with different concentrations of D-glucose plus 5-^3^H-glucose and U-^14^C glucose. Perfusate samples were collected every 20 min for LAC measurement. Labeled LAC concentrations were derived from U-^14^C glucose; unlabeled LAC concentrations were derived from endogenous substrates such as amino acids (Kerr et al., 1979). In **(B)**, total LAC production is plotted as a function of recirculation time. In **(C)**, exogenous, endogenous, and total LAC production rates are plotted separately as a function of buffer GLC concentration. Values are mean ± SE (*n* = 6 or more for each GLC concentration). Lines are model fits to data. **(D)** Effect of perfusate LAC concentration on lung GLC consumption rate (Fisher and Dodia, 1984). Lungs were isolated from male Sprague-Dawley rats (180–220 g) and perfused with perfusate containing 10 mM 5-^3^H-glucose and D-glucose. In addition, different concentrations of LAC were added to perfusate before recirculation. ^3^H_2_O production rate was measured as an index of GLC consumption rate. 0.5 mM LAC had no effect on GLC consumption rate, but as buffer LAC concentration increased to 2 mM, GLC consumption rate decreased to half as compared to that under baseline conditions (no LAC). Symbols are mean of values from 4 to 10 lungs, and lines are model fits to data.

**Figure 4:**
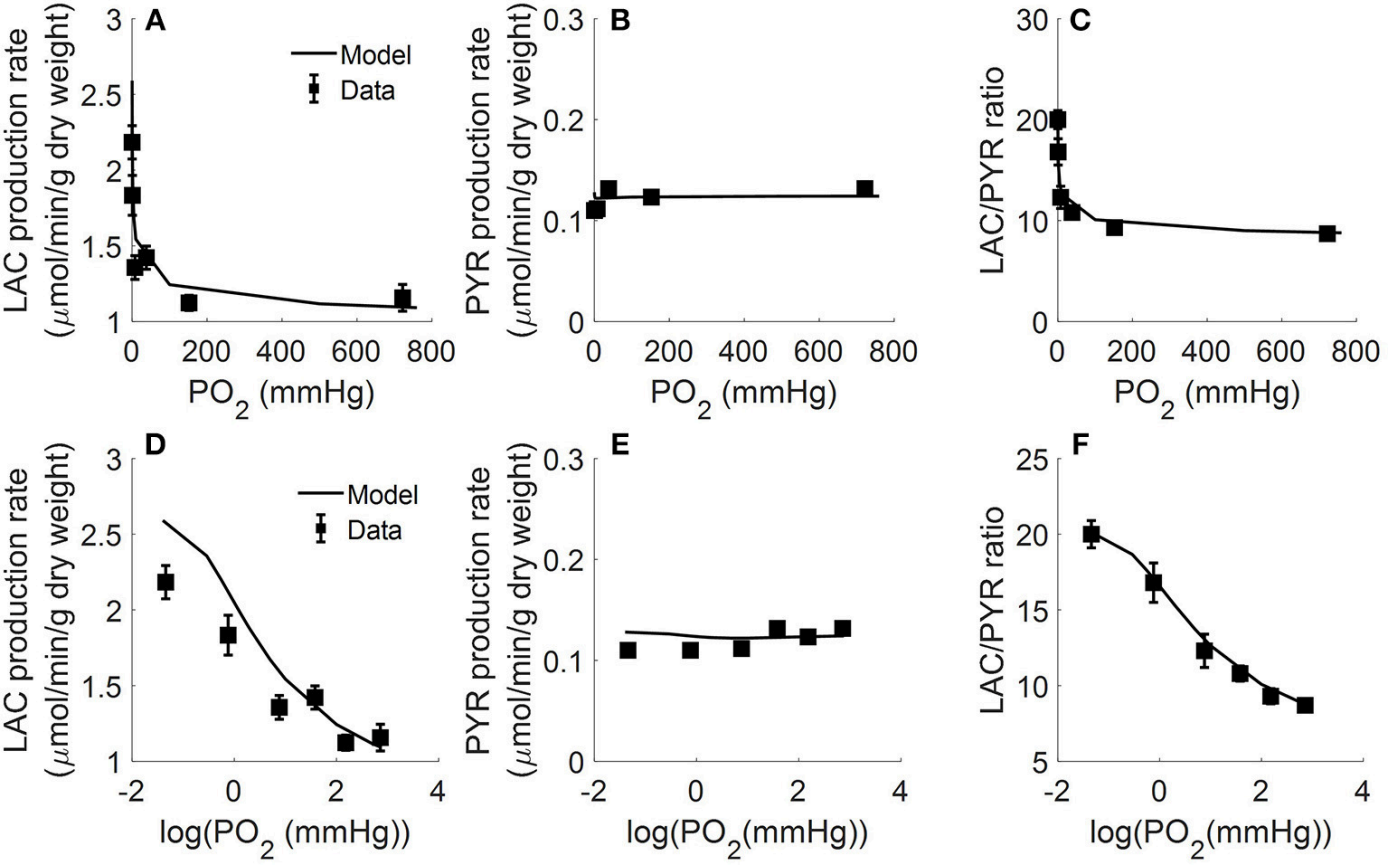
Lungs were isolated from male Sprague-Dawley rats (200–250 g) and perfused with 40 ml Krebs-Ringer bicarbonate buffer containing 10 mM glucose (GLC) and 3% bovine serum albumin (Fisher and Dodia, 1981). Gas mixtures containing different % oxygen partial pressures (PO_2_; ranging from 0.006 to 95%) were used to ventilate the lungs and aerate the perfusate. Perfusate sampled were collected for lactate (LAC) and pyruvate (PYR) measurements. Rat lung LAC production rate **(A,D)**, PYR production rate **(B,E)**, and LAC/PYR ratio **(C,F)** are plotted as a function of %PO_2_ (top panels) or log (%PO_2_) (bottom panels) in gas mixture to show the model fit to data at low %PO_2_ (Fisher and Dodia, 1981). Symbols are experimental data (Fisher and Dodia, 1981) and solid lines are model fits.

For the mitochondria matrix and IMS regions, the values of intrinsic (apparent Michaelis constants, *K'*s) and all extrinsic model parameters (*V*_*maxf*_*s* and *T*_*maxf*_*s*), except for the parameter descriptive of mitochondrial membrane leakiness, were fixed to those estimated using our model of isolated lung mitochondria bioenergetics (Zhang et al., 2018). For these regions, the values of the extrinsic model parameters, expressed in units of nmol/min/mg mitochondria protein in the isolated lung mitochondria model, were converted to nmol/min/lung for the integrated lung model by multiplying each parameter by total mitochondria protein mass (*M*_*mito*_) in the rat lung. The value of *M*_*mito*_ along with those for other unknown model parameters (Table 2) were estimated from the experimental data (Figures 2–4) described below. For the lung model, the value of *T*_*maxf, LEAK*_,the parameter descriptive of mitochondrial membrane leakiness, was assumed to be equal to or lower than that estimated from isolated mitochondria (Zhang et al., 2018), since the mitochondria isolation process could cause damage to mitochondria membrane. Thus, *T*_*maxf, LEAK*_was assumed to be an unknown parameter for the lung model (Table 2).

**Table 2:** Estimated values of unknown model parameters (30°C).

**Parameters**	**Definition**	**Parameter value (nmol/min/lung)**	Experimentally measured value (nmol/min/lung) Pérez-Díaz et al., 1977*
**GLYCOLYSIS PATHWAY REACTIONS**			
*V_*maxf, HK*_*	Maximum forward reaction rate of HK	279	705 ± 93.1
*V_*maxf, PGI*_*	Maximum forward reaction rate of PGI	3.70 × 10^4^	4.2 × 10^4^ ± 3.7 × 10^3^
*V_*maxf, PFK*_*	Maximum forward reaction rate of PFK	1.47 × 10^3^	8.3 × 10^3^ ± 1.2 × 10^3^
*V_*maxf, ALD*_*	Maximum forward reaction rate of ALD	1.43 × 10^3^	5.72 × 10^3^ ± 146
*V_*maxf, GAPDH*_*	Maximum forward reaction rate of GAPDH	6.35 × 10^4^	5.53 × 10^3^ ± 864
*V_*maxf, PGK*_*	Maximum forward reaction rate of PGK	2.00 × 10^5^	8.15 × 10^4^ ± 1 × 10^4^
*V_*maxf, PK*_*	Maximum forward reaction rate of PK	1.89 × 10^4^	1.83 × 10^4^ ± 784
*V_*maxf, LDH*_*	Maximum forward reaction rate of LDH	3.92 × 10^5^	4.74 × 10^4^ ± 5.6 × 10^3^
**OTHER PARAMETERS**			
*V_*maxf, G*6*PDH*_*	Maximum forward reaction rate of G6PDH	2.82 × 10^3^	NA
*V_*maxf, ATPase*_*	Maximum forward reaction rate of ATPase	2.54 × 10^4^	NA
*V_*maxf, AA*_*	Maximum forward reaction rate of AA	409	NA
*T_*maxf, GLUT*_*	Maximum transport rate of GLUT	534.12	NA
*T_*maxf, PYRT*_*	Maximum transport rate of PYRT	185.08	NA
*T_*maxf, LACT*_*	Maximum transport rate of LACT	6.36 × 10^3^	NA
*T_*maxf, MAS*_*	Maximum transport rate of MA shuttle	4.43 × 10^3^	NA
*T_*maxf, LEAK*_*	Maximum rate of passive proton leak	15.98	NA
*M_*mito*_*	Average mass of mitochondria protein in rat lungs	16.44 mg	13.12 mg**

* *values are converted from μmol/g wet weight/min to nmol/min/lung*.

** *Mitochondrial protein content is 1 mg/1 μL mitochondrial volume*.

*NA, Not available. Values are mean ± SE*.

For the cytosolic region, the values of the intrinsic model parameters, such as apparent Michaelis constants (*K'*s) of various substrates and products for different enzymes and transporters, were set to previously published values (see Supporting Information). The assumption is that since the apparent Michaelis constants are intrinsic model parameters their values are organ-independent (Zhang et al., 2018). For a given enzymatic or transport reaction, the MATLAB optimization toolbox function “*fmincon*” was used to estimate the intrinsic parameters by fitting the reaction flux to pertinent data (see Supporting Information). The values of the extrinsic model parameters (i.e., *V*_*maxf*_s, *T*_*maxf*_s) in Table 2 were estimated by fitting the model solution to experimental data (Figures 2–4) from rat lung tissue bioenergetics using the MATLAB optimization toolbox function “*ga*.” This function implements a genetic algorithm, which is a derivative-free optimization algorithm (Rios and Sahinidis, 2013) suitable for large-scale metabolic models. For the “*ga*” function, the objective function *E* optimized was:

(8) $$\begin{array}{l} E=\frac{1}{N}{\overset{M}{\underset{j=1}{\sum }}\overset{N}{\underset{i=1}{\sum }}\left(\frac{x_{i,j}-X_{i,j}}{X_{i,j}}\right)}^{2} \end{array}$$

where *x*_*i, j*_ and *X*_*i, j*_ are the model solutions and the corresponding experimental data at the *i*^*th*^ time point and *j*^*th*^ data set, respectively. *N* is the number of data points and *M* is the number of data sets used for the parameter estimation. Experimentally measured maximal enzyme activities (Table 2) were used as initial guesses for the *V*_*maxf*_values of corresponding reactions in the glycolysis pathway (Pérez-Díaz et al., 1977). A total of 17 unknown extrinsic parameters (Table 2) were estimated from the experimental data in Figures 2–4 (Kerr et al., 1979; Fisher and Dodia, 1981, 1984; Fisher, 1984; Bongard et al., 2013).

## Results

### Experimental Data Used for Estimating the Unknown Model Parameters (Table 2)

The following existing experimental data sets (Figures 2–4) were used to estimate the values of the unknown model parameters (i.e., maximal reaction and transport velocities) related to the cytosolic reactions and plasma membrane metabolite transporters (Table 2).

Figure 2A shows the rates of glucose consumption rate (Kerr et al., 1979), and the rates of production of pyruvate and total lactate (Fisher and Dodia, 1981), for perfusate glucose concentration of 5.5 mM. The data in Figure 2B are the pseudo-steady state reaction flux of the pentose phosphate pathway (Fisher, 1984). Audi et al. estimated the reaction flux of mitochondrial complex IV as the cyanide-sensitive oxygen consumption rate (OCR) calculated as the difference in isolated rat lung OCR measured in the absence vs. presence of potassium cyanide (complex IV inhibitor, 2 mM) (Figure 2C) (Audi et al., 2003). The data in Figure 2D show the rat lung cytosolic and mitochondrial ATP production rates estimated by Fisher et al. based on glucose-carbon recovery from glucose (Fisher, 1984).

Kerr et al. evaluated the effect of perfusate glucose concentration on the glucose consumption rate as well as lactate production rate in isolated perfused rat lungs (Kerr et al., 1979). The lungs were perfused with different concentrations of D-glucose along with labeled glucose (5-^3^H-glucose and U-^14^C glucose, each with specific activity of 0.1 mCi/mmol). The labeled glucose forms were used to measure lung glucose consumption rate, and to distinguish lactate production rate from glucose added to the perfusate (exogenous source) from that derived from endogenous substrates such as amino acids and glycogen (Kerr et al., 1979). Following the lung uptake of 5-^3^H-glucose, tritium (^3^H) is liberated during glycolysis to form ^3^H_2_O. Over 90% of the ^3^H from glucose was recovered as ^3^H_2_O (Kerr et al., 1979). As such, the production rate of ^3^H_2_O was used as a measure of lung glucose consumption rate (Kerr et al., 1979). For a given perfusate sample, ^3^H_2_O was separated from 5-^3^H-glucose by evaporation of the sample (Bassett and Fisher, 1976a). ^14^C labeled lactate measured in perfusate was derived from U-^14^C glucose and is referred to as exogenous lactate since it is derived from exogenous sources (i.e., glucose added to the perfusate). Lactate derived from U-^14^C glucose was separated from U-^14^C glucose by extraction with ether (Kerr et al., 1979). Endogenous lactate in a given perfusate sample was then obtained as the difference between total lactate in the sample, measured enzymatically, and labeled lactate (Kerr et al., 1979). The resulting data are shown in Figures 3A–C.

The data in Figure 3 show the amount of glucose consumed Figure 3A and total lactate produced Figure 3B as a function of recirculation time over a range of perfusate glucose concentrations. The results in panel C show a nonlinear relationship between the perfusate glucose concentration and the lung rate of glucose consumption, consistent with the saturability of glucose transporters (Kerr et al., 1979). The results in Figure 3C also show that the rate of endogenous lactate production is saturable at low perfusate glucose concentrations, whereas the rate of exogenous lactate production is relatively linear over the range of perfusate glucose concentrations studied. In the model, exogenous (*LAC*_*exo*_) and endogenous (*LAC*_*endo*_) lactate production rates were calculated as

(9a) $$\begin{array}{lll} LAC_{endo} & = & \frac{J_{AA}}{J_{PK}+J_{AA}}J_{LACT} \end{array}$$

(9b) $$\begin{array}{lll} LAC_{exo} & = & \frac{J_{PK}}{J_{PK}+J_{AA}}J_{LACT} \end{array}$$

where *J*_*AA*_, *J*_*PK*_ and *J*_*LACT*_are the reaction/transport rates of alanine aminotransferase, pyruvate kinase, and lactate transport between blood and cytosol, respectively.

Fisher and Dodia evaluated the effect of perfusate lactate concentration on lung glucose consumption rate in isolated rat lungs perfused with perfusate containing 5-^3^H-glucose and different D-glucose concentrations (Fisher and Dodia, 1984). ^3^H_2_O production rate was measured as an index of glucose consumption rate. The data in Figure 3D show that 0.5 mM lactate had no effect on glucose consumption rate, but as perfusate lactate concentration increased to 2 mM, the glucose consumption rate decreased to half that measured under baseline conditions (zero exogenous lactate). These data suggest that exogenous lactate has a regulatory effect on rat lung glycolytic rates (Wolfe et al., 1979; Fisher and Dodia, 1984;Wang et al., 2004).

In another study, Fisher and Dodia measured the production rates of lactate and pyruvate in isolated perfused rat lungs as a function of O_2_ partial pressure (PO_2_) in lung ventilation/aeration gas mixture (Fisher and Dodia, 1981). Gas mixtures containing %PO_2_ ranging from 0.006 to 95% were used to ventilate the lungs and aerate the perfusate. Perfusate samples were then collected for lactate and pyruvate measurements. Figure 4 shows that pyruvate production rate was not significantly altered over the range of PO_2_ studied. Lactate production rate also was not significantly altered for PO_2_ > 0.1% (0.76 mmHg) (Fisher and Dodia, 1981). However, the lactate production rate increased by ~40 and ~80% for PO_2_ levels of 0.1 and 0.006%, respectively. These results suggest that the rat lung can maintain normal metabolism for PO_2_ ≥ 0.1% (Fisher and Dodia, 1981).

All of the extrinsic parameters of the model (Table 2) were estimated by simultaneously fitting the model solution to the experimental data in Figures 2–4 using the genetic algorithm as above. Results are shown in Table 2, and bars in Figure 2 and the lines superimposed on the data in Figures 3, 4 are the model fits.

### Measures of Identifiability and Estimability of the Extrinic Parameters of the Model

To assess the identifiability and estimability of the extrinsic parameters of this lung tissue bioenergetics model (Table 2), we estimated the parameters' normalized sensitivity coefficients and a matrix of correlation coefficients between the estimated model parameters. The normalized sensitivity coefficients provide information about the contribution of each of the extrinsic model parameters to the overall model solution, whereas the correlation coefficient matrix provides information about the degree of interdependence between the various model parameters. For a given parameter, the normalized sensitivity coefficient was determined using Equation 10 (Zhang et al., 2018):

(10) $$\begin{array}{l} S_{\theta_{i}}=\frac{\theta_{i}}{E}\left(\frac{\partial E}{\partial \theta_{i}}\right) \end{array}$$

where *E* is the sum of squared difference between experimental data (Figures 2–4) and the model fit, as defined by Equation 8, and θ_*i*_ is the estimated value of *i*^th^ extrinsic model parameter. $\frac{\partial E}{\partial \theta_{i}}$ was approximated using the central difference method with 0.1% change in θ_*i*_.

The matrix of correlation coefficients between the model parameters was evaluated at the estimated parameter values in Table 2 that best fit the model to the available experimental data. The correlation coefficient (*CC*_*ij*_) between the *i*th parameter and *j*th parameter was determined using Equation 11 (Audi et al., 2001):

(11) $$\begin{array}{l} CC_{ij}=\frac{HH_{ij}}{\sqrt{HH_{ii}*HH_{jj}\;}}\;fori,j=1,\;\ldots ,np\; \end{array}$$

where *np* is the number of model parameters, *HH* is the inverse of the product of the transpose of the Jacobian matrix and the Jacobian matrix evaluated at the estimated values of the model parameters in Table 2 that best fit the model to the data in Figures 2–4.

**Figure 5:**
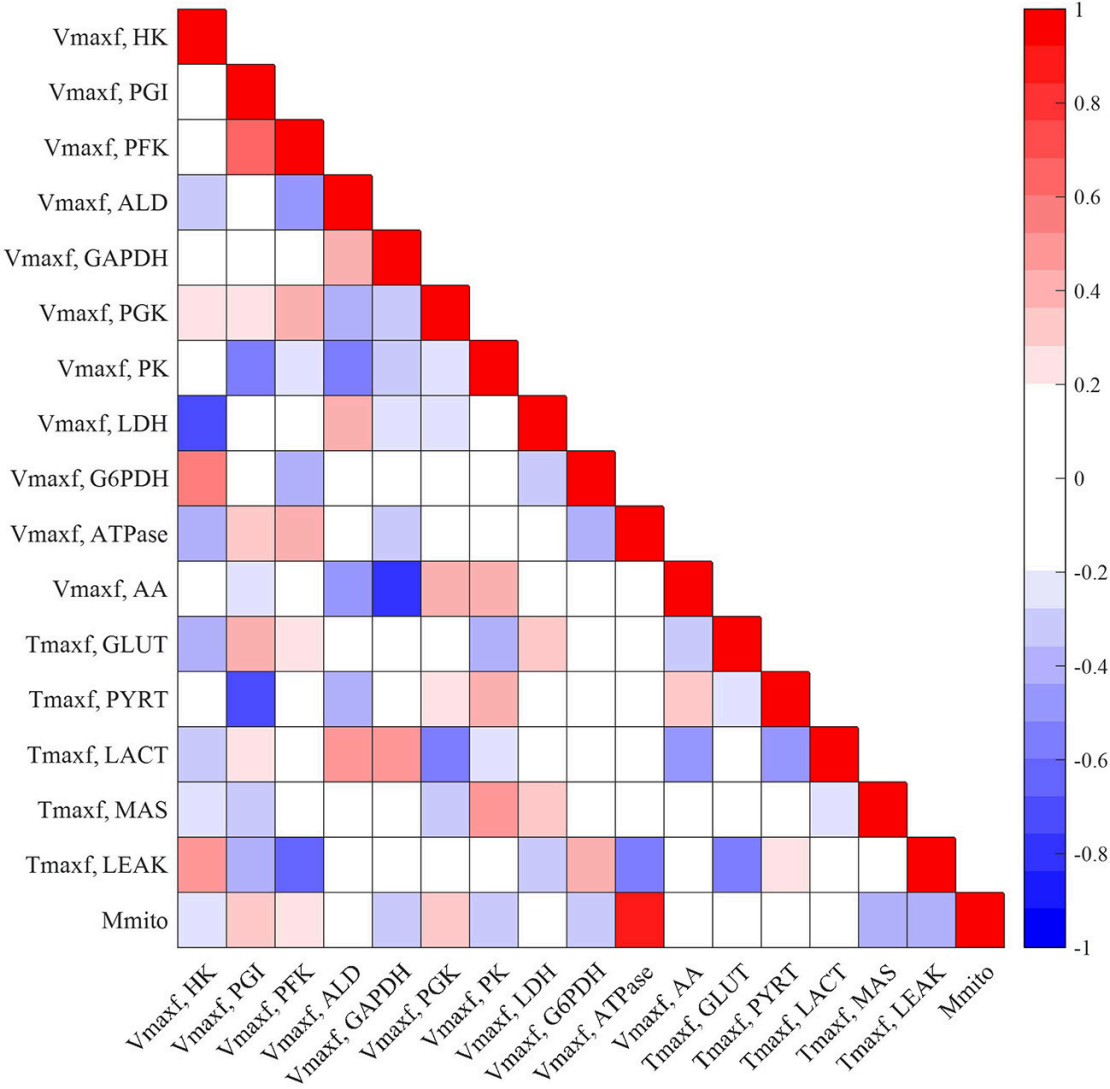
Matrix of correlation coefficients between the extrinsic parameters of the model. Correlation coefficients range between −1 (perfect negative correlation) and +1 (perfect positive correlation) and are estimated using Equation (11). A small positive or negative correlation coefficient between two parameters suggests small interdependence between those parameters.

**Figure 6:**
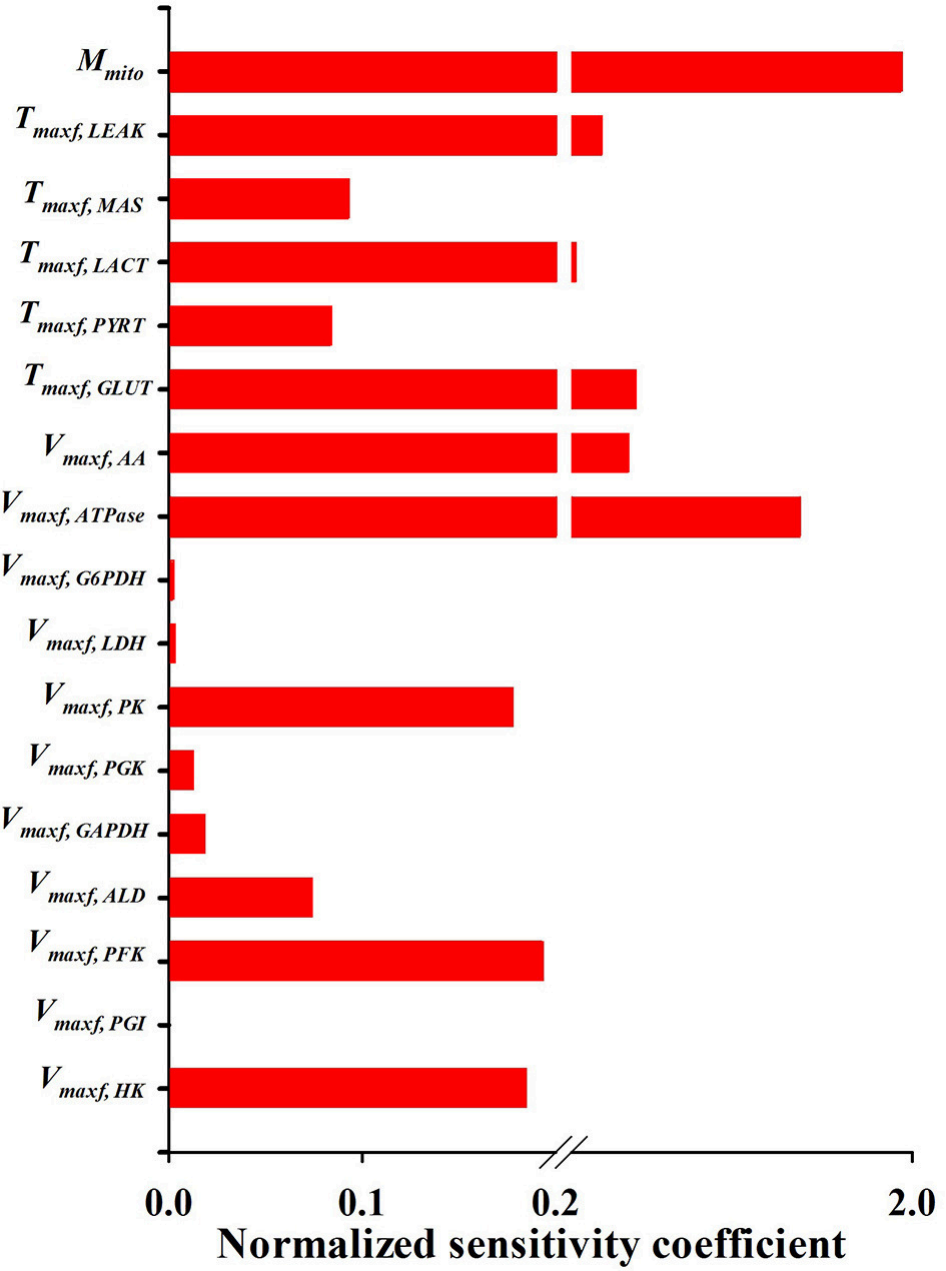
Normalized sensitivity coefficients of the extrinsic model parameters. A parameter contribution to the model solution is proportional to its normalized sensitivity coefficient estimated using Equation (10).

Figures 5, 6 show the respective normalized sensitivity coefficients and matrix of correlation coefficients for the extrinsic model parameters. For most of the extrinsic model parameters, the normalized sensitivity functions are relatively high, and the correlation coefficients are relatively low, consistent with a tight range of values for those parameters that provide a good fit to the data in Figures 2–4. This suggests that the chosen experimental data has enough information to estimate the values of the unknown model parameters.

### Model Validation

**Figure 7:**
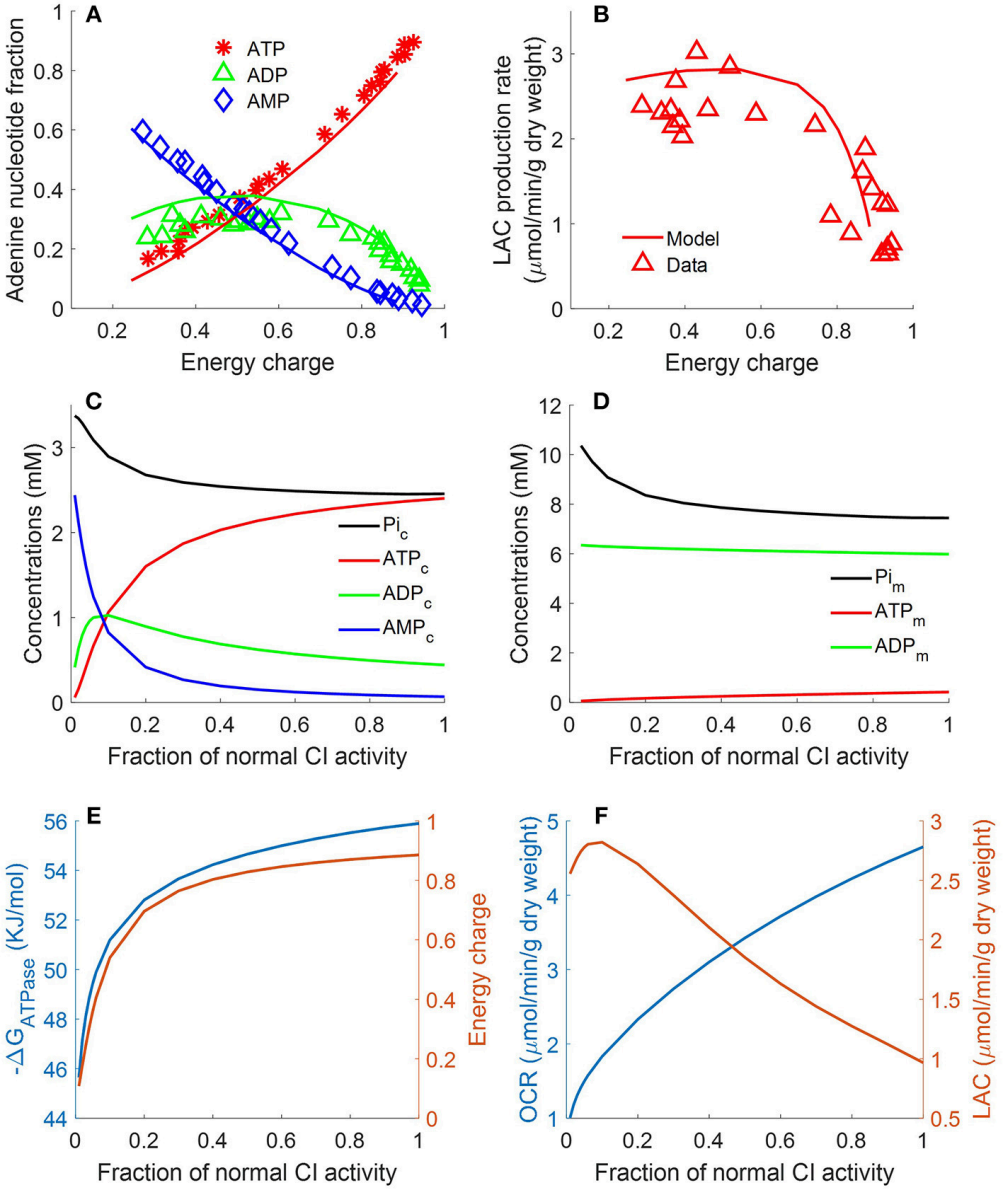
Lung fractional ATP, ADP, and AMP contents **(A)**, lung lactate (LAC) production rate **(B)** as functions of energy charge (*EC*) defined in Equation 12 (Bongard et al., 2013). For the data (symbols) in **(A,B)**, lungs were isolated from male Sprague-Dawley rats (*n* = 36) and perfused with perfusate containing 5.5 mM glucose and 5% bovine serum albumin at 37°C. Isolated perfused rat lungs were exposed to single or combination of different treatments, including 20 μM rotenone (complex I inhibitor), 50 μM CoQ_1_ (reduced to CoQ_1_H_2_, an artificial electron donor), or 3.6 μM antimycin A (complex III inhibitor).These treatments induced different degrees of change in lung *EC*, adenine nucleotides contents, and LAC production rates. For model predictions (solid lines), mitochondrial complex I activity was incrementally decreased to simulate the inhibitor effects of rotenone on lung EC. In **(A)**, lung ATP amount decreased as *EC* decreased, while AMP amount increased as *EC* decreased. Lung ADP amount showed a biphasic behavior. In **(B)**, lung LAC production rate increased as lung EC decreased. **(C)** Shows model predicted relationships between CI activity and cytosolic concentrations of ATP, ADP, AMP, and Pi. **(D)** Shows model predicted relationships between complex I (CI) activity and mitochondrial concentrations of ATP, ADP, and phosphate (Pi). **(E)** Shows model predicted relationships between CI activity and *EC* and cytosolic ATP hydrolysis potential (Δ_*r*_*G*_*ATPase*_). **(F)** Shows model predicted relationships between CI activity, lung oxygen consumption rate (OCR) and lung lactate (LAC) production rate.

**Figure 8:**
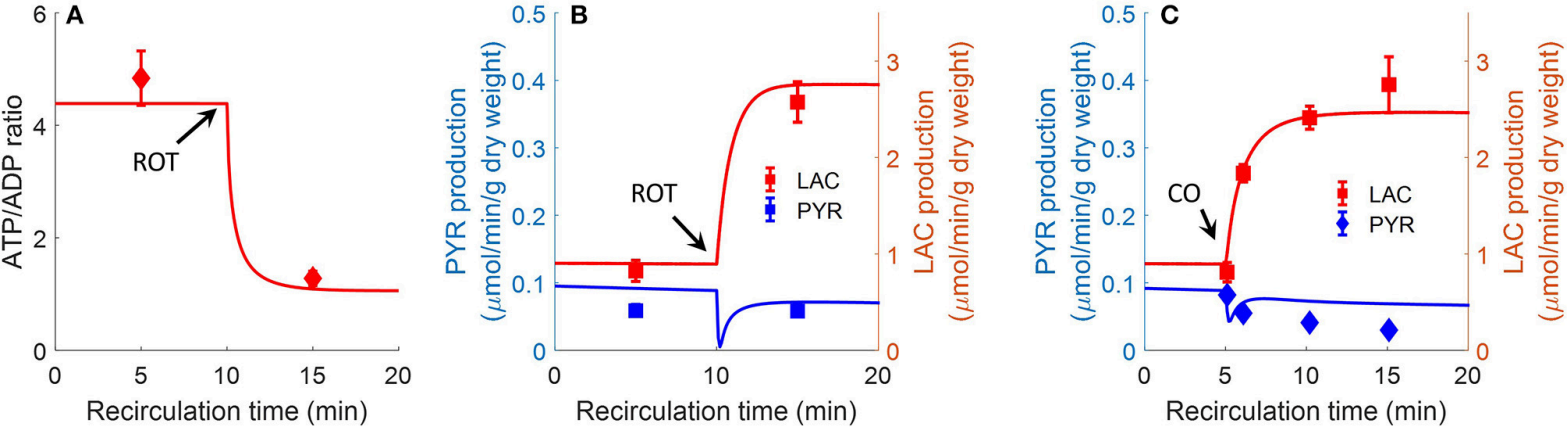
Effect of complex I inhibitor rotenone (ROT, 20 μM) on lung ATP/ADP **(A)** ratio and LAC, PYR production rates **(B)** in perfusate recirculated through isolated perfused rat lungs (Bongard et al., 2013). Experimental conditions are the same as those for data in Figure 7. For model predictions, ROT effect was simulated by decreasing mitochondrial Complex I activity by 85% at 10 min, with all other parameters (Table 2) unchanged. Symbols are experimental data (Bongard et al., 2013), values are mean ± SE (*n* = 4). Solid lines are model predictions. **(C)**. Effect of complex IV inhibitor CO (carbon monoxide) on lung LAC, PYR production rates. At control condition, the lung was ventilated with 95% O_2_ and 5% CO_2_. At 5 min, the ventilation gas was changed to 95% CO and 5% CO_2._ For model predictions, CO effect was simulated by decreasing mitochondria complex IV activity by 99.7%. Symbols are experimental data (Fisher et al., 1976) and solid lines are model predictions. LAC production rate was multiplied by a factor of 2 since LAC production measured in Fisher et al. (1976) is 50% lower than in Bongard et al. (2013).

**Figure 9:**
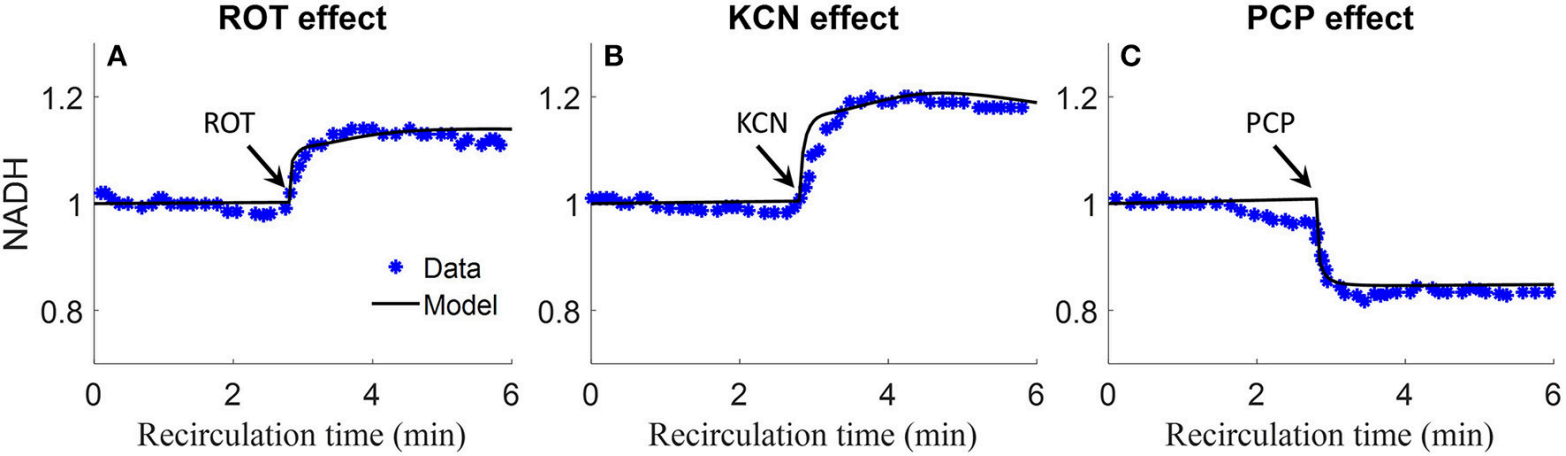
Experimental data and model predictions of normalized lung surface NADH (Sepehr et al., 2013; Staniszewski et al., 2013). Lungs were isolated from male Sprague-Dawley rats (300–350 g) and perfused with perfusate containing 5.5 mM glucose and 3% bovine serum albumin at 37°C. NADH fluorescence intensity was measured using fluorometer with fiber optic probe placed against the pleural surface of right lobe. Signals were first obtained under resting condition, and then following the addition of inhibitors to the recirculating perfusate to induce changes in NADH redox status and thus fluorescence intensities. For **(A–C)**, 20 μM rotenone (ROT, complex I inhibitor), 2 mM potassium cyanide (KCN, complex IV inhibitor), or 3 mM protonophore (PCP, uncoupler) was added to the recirculating perfusate at 3 min, respectively. For model predictions, ROT effect was simulated by decreasing mitochondrial Complex I activity by 85%, KCN effect was simulated by decreasing mitochondria complex IV activity by 99.7%, and PCP effect was simulated by increasing proton leak activity by 5 times. Symbols are experimental data (Sepehr et al., 2013; Staniszewski et al., 2013), and solid lines are model predictions.

To validate or corroborate the model, we assessed its ability to predict published experimental data that were not used for model development, including estimating the values of the unknown model parameters. To that end, we evaluated the ability of the model to predict the experimental data in Figures 7–9 and Table 3.

**Table 3:** Model predicted and experimental lung tissue adenine nucleotide fractions and content.

	**Model predicted**	**Bongard et al., 2013**	**Akai et al., 1998**	**Fisher, 1978**
ATP/(ATP+ADP+AMP)	0.78	0.79	0.83	0.83
ADP/(ATP+ADP+AMP)	0.19	0.16	0.12	0.12
AMP/(ATP+ADP+AMP)	0.03	0.04	0.05	0.05
ATP (μmole/g dry wt)	6.64	5.66 ± 0.46	6.8 ± 0.5	12.8 ± 0.1
ADP (μmole/g dry wt)	1.85	1.17 ± 0.14	0.95 ± 0.05	1.8 ± 0.2
AMP (μmole/g dry wt)	0.29	0.31 ± 0.06	0.41 ± 0.02	0.8 ± 0.1

*For lung tissue content of adenine nucleotides, experimental values are mean ± SE*.

Bongard et al. measured fractional ATP, ADP, and AMP contents in isolated perfused rat lungs and calculated the energy charge (*EC*).

(12) $$\begin{array}{l} EC=\frac{ATP+0.5ADP}{ATP+ADP+AMP} \end{array}$$

following exposure to a single or combination of treatments, including rotenone (complex I inhibitor, 20 μM), ubiquinol analog coenzyme Q1 (oxidized artificial electron donor, 50 μM), or antimycin A (complex III inhibitor, 3.6 μM) (Bongard et al., 2013). These treatments induced different degrees of change in lung *EC*, adenine nucleotides contents, and lactate production rates. Figure 7A shows that lung ATP content decreased as *EC* decreased, whereas AMP content increased as *EC* decreased. Lung ADP content shows a biphasic behavior as a function of *EC*. As shown in Figure 7B, lung lactate production rate increased as lung EC decreased. However, when *EC* dropped below 0.4, lung lactate production rate did not increase further. The two glycolytic enzymes HK and PFK require ATP as substrate. Model simulations show that when *EC* falls below 0.4, ATP content becomes too low for these two enzymes to proceed. Therefore, under such conditions, most of the lactate produced by the rat lung is from endogenous substrates such as alanine. As shown in Figure 7, model predictions using the estimated values of the model parameters in Table 2 are in good agreement with the measured experimental data. For model predictions, the model parameters descriptive of the activity of mitochondrial complex I was gradually decreased to simulate the inhibitory effects of rotenone on *EC*. Additional model simulations in Figure 7C show that as cytosolic ATP concentration decreases (due to decrease in complex I (CI) activity), cytosolic phosphate (Pi) concentration as well as cytosolic ADP + AMP increase. Model simulations in Figure 7D show mitochondrial concentration of ATP, ADP, and Pi as a function of complex I activity.

The ATP hydrolysis potential, Δ_*r*_*G*_*ATPase*_, is a measure of the energy released by ATP hydrolysis and is more thermodynamically relevant than *EC* (Erecinska et al., 1977). We calculated Δ_*r*_*G*_*ATPase*_ using the following equation:

(13) $$\begin{array}{l} \Delta_{r}G_{ATPase}=\Delta_{r}G_{ATPase}^{0}+\left(RT\right)ln\left(\frac{\left[ADP_{c}\right]\left[Pi_{c}\right]}{\left[ATP_{c}\right]}\right) \end{array}$$

where [*ATP*_*c*_], [*ADP*_*c*_], and [*Pi*_*c*_] are concentrations of ATP, ADP, and inorganic phosphate (Pi) in the cytosolic region, respectively; Δ_*r*_G${}_{ATPase}^{0}$, *R* and *T* are the standard Gibbs free energy of the reaction at pH = 7, gas constant, and temperature, respectively. Model simulations in Figures 7E,F show that decreasing CI activity leads to decrease in lung *EC*, decrease in cytosolic Δ_*r*_*G*_*ATPase*_, decrease in lung O_2_ consumption rate (OCR), and increase in lung rate of lactate (LAC) production. The simulations in Figure 7E suggests a high correlation between *EC* and Δ_*r*_*G*_*ATPase*_, consistent with theoretical calculations by (Erecinska et al., 1977).

Bongard et al. also evaluated the effect of the complex I inhibitor rotenone (20 μM) on lung ATP/ADP ratio and on lactate/pyruvate ratio in perfusate recirculated through isolated perfused rat lungs (Bongard et al., 2013). As shown in Figure 8, model simulations are in good agreement with the measured data. For model predictions, the rotenone inhibitory effect was simulated by decreasing mitochondrial complex I activity by 85%, with the values of all the other model parameters set to those in Table 3. The carbon monoxide (CO) inhibitory effect on lung mitochondrial function was simulated by decreasing mitochondria complex IV activity by 99.7% at 10 min, with the values of all the other model parameters set to those in Table 2.

Table 3 shows experimentally measured (Bongard et al., 2013) and model predicted lung tissue adenine nucleotide fractions and contents under control conditions. Model predictions are within the range of measured experimental data.

Staniszewski et al. and Sepehr et al. measured lung surface NADH (top panels) emission signal in isolated perfused rat lungs by placing a fiber optic probe against the pleural surface of the right lobe (Sepehr et al., 2013; Staniszewski et al., 2013). Signals were first obtained under resting condition, and then following the addition of inhibitors to the recirculating perfusate to induce changes in NADH redox status, and thus fluorescence intensities. As shown in Figure 9, rotenone (ROT, complex I inhibitor) caused a 20% increase in NADH intensity. Potassium cyanide (KCN, complex IV inhibitor) caused 20% increase in NADH intensity. The mitochondrial uncoupler pentachlorophenol (PCP) caused a 20% decrease in NADH intensity. For model predictions, the ROT effect was simulated by decreasing mitochondrial complex I activity by 85%, KCN effect was simulated by decreasing mitochondria complex IV activity by 99.7%, and PCP effect was simulated by increasing the proton leak activity (parameter *T*_*maxf, LEAK*_) by 5 times. As shown in Figure 9, model simulations are in good agreement with the measured experimental data.

## Discussion

We developed and validated the first integrated computational model of the bioenergetics of intact rat lungs. The model validation involved determining its ability to predict well a wide range of published experimental data that were not used for the development of the model. The model, which is an extension of our previously developed integrated computational model of the bioenergetics of isolated rat lung mitochondria (Zhang et al., 2018), provides important insights into lung mitochondrial and tissue bioenergetics, and allows us to predict system behavior and changes in important system properties that are either experimentally testable or technically difficult to measure.

### Parameter Estimation Results and Sensitivity Analysis

As shown in Figures 2–4, the model was able to fit a wide range of published experimental data collected from isolated perfused rat lungs under various experimental conditions, including metabolic changes in response to different buffer glucose concentrations, lactate concentrations, oxygen partial pressures, as well as the effects of various metabolic inhibitors.

Except for the maximum mitochondria proton leak activity (*T*_*maxf, LEAK*_), the values of the model parameters for the mitochondria matrix and IMS regions were set to those estimated using our recent model of the bioenergetics of mitochondria isolated from rat lungs (Zhang et al., 2018). Our estimate of *T*_*maxf, LEAK*_ from isolated mitochondria ranged between ~15 nmol/min/mg and 36 nmol/min/mg mitochondria protein, depending on the quality of the isolated mitochondria, since the isolation process could damage the mitochondrial membrane (Zhang et al., 2018). For the whole lung, the value of *T*_*maxf, LEAK*_ would be expected to be equal to or smaller than that estimated from isolated mitochondria (Zhang et al., 2018). For the lung model, the estimated value of *T*_*maxf, LEAK*_was ~16 nmol/min/mg mitochondria protein which is close to the low end of the range of values estimated from isolated mitochondria (Zhang et al., 2018).

For glycolytic enzymes, we used experimentally measured enzyme activities (Pérez-Díaz et al., 1977) as initial guesses for the unknown extrinsic model parameters (Table 2), which significantly improved the efficiency of the genetic algorithm used for parameter estimation. Optimal parameter estimates obtained using experimental data in Figures 2–4 show that most of the estimated *V*_*maxf*_ values are relatively close to the measured activities of the corresponding enzymes (Table 2). This, along with the ability of the model to predict quite well additional experimental data (Figures 7–9, Table 3) that were not used for model parameter estimation, serves as validation of this rat lung bioenergetics model.

An activity parameter (i.e., *V*_max_'s or *T*_max_'s) with a relatively high normalized sensitivity coefficient suggest that the enzyme or transporter described by this parameter is likely to be the rate limiting step, since a small change in that parameter will result in relatively large change in the model output. Model sensitivity analysis revealed key information regarding the limiting step(s) in glycolysis. Consistent with enzyme studies in isolated rat lung cells (Pérez-Díaz et al., 1977), few glycolytic enzyme activities greatly exceeded the observed rate of glucose utilization. As such, the calculated normalized sensitivity coefficients (Figure 6) are low for the glycolytic enzyme activity model parameters, including phosphoglucose isomerase (*PGI*) and phosphoglycerate kinase (PGK). On the other hand, glucose transporter (*GLUT*), phosphofructokinase (*PFK*), and hexokinase (*HK*) have relatively high sensitivity coefficients, and hence are probably the glycolysis rate limiting steps in lung tissue.

**Figure 10:**
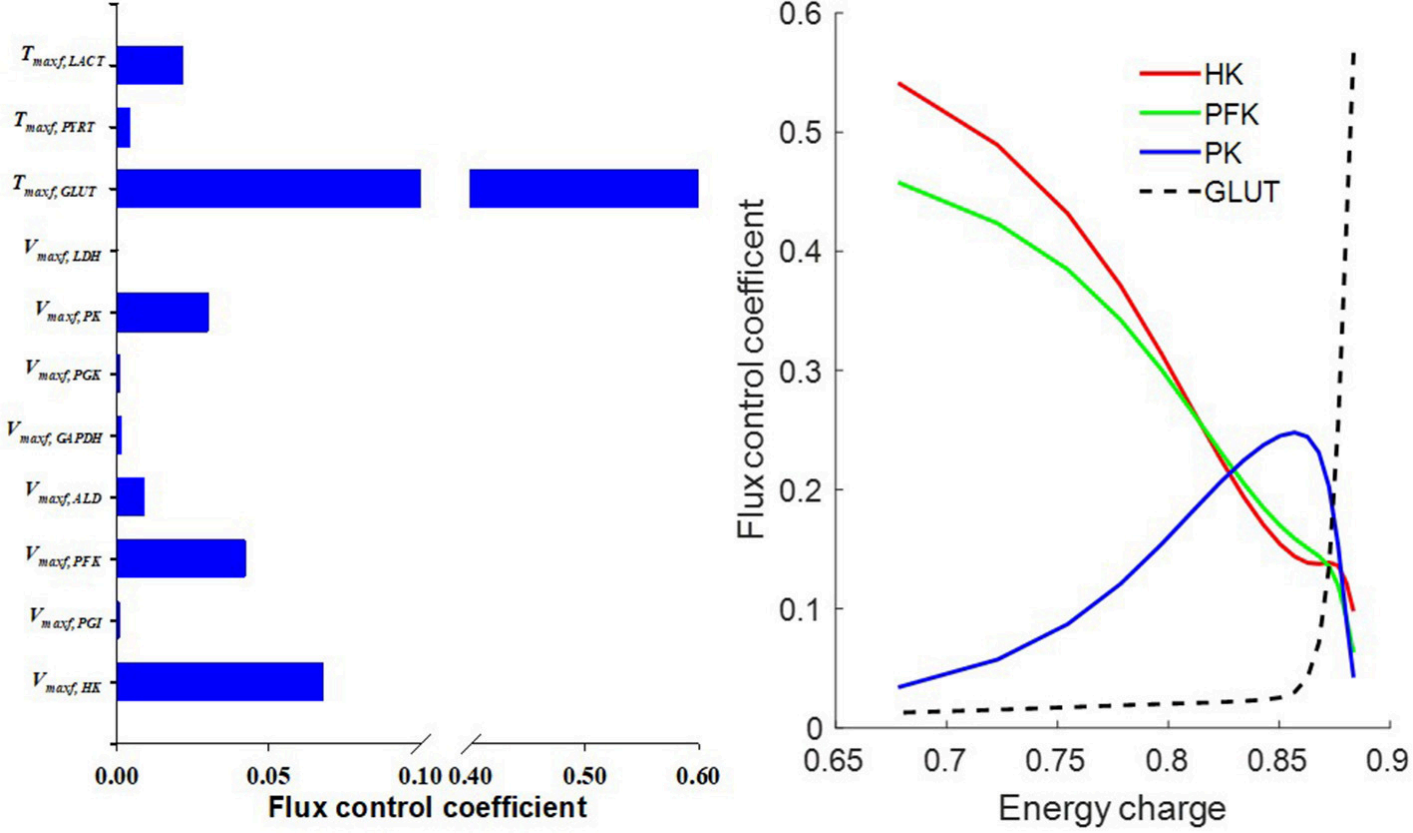
**(Left)** Model predicted flux control coefficients for glycolytic enzymes and for the transporters that are affected by glycolysis under basal conditions. **(Right)** Flux control coefficients as a function of energy charge (*EC*, defined in Equation 12). The flux control coefficient of the glucose transporter (*GLUT*) decreased as *EC* decreased, whereas flux control coefficients of the glycolytic enzymes phosphofructokinase (*PFK*) and hexokinase (*HK*) increased as *EC* decreased. For model simulations, mitochondrial complex I activity was incrementally decreased to induce a decrease in *EC*.

Flux control coefficient for a given enzyme or transporter is defined as the relative change in a pathway's flux (e.g., glycolysis) with respect to a change in the value of a model parameter descriptive of the activity of that enzyme or transporter in that pathway (Fell, 1997). To gain more insights into the rate limiting step for glycolysis, we calculated the flux control coefficients (Fell, 1997; Liguzinski and Korzeniewski, 2006) for glycolytic enzymes and for the transporters that are affected by glycolysis. The sum of those flux control coefficients should be one, as predicted by the metabolic control theory (Liguzinski and Korzeniewski, 2006). The results shown in Figure 10 (left panel) suggest that under basal condition, *GLUT* has the highest flux control coefficient, and hence is the rate limiting step for glycolysis. However, Figure 10 (right panel) shows that when lung *EC* decreases, the flux control coefficients of the glycolytic enzymes *HK* and *PFK* increase significantly since they use ATP as a substrate, whereas that for *GLUT* decreases. Thus, under conditions of low *EC, HK*, and PFK are potentially the rate limiting steps for glycolysis.

### Differences Between the Bioenergetics of Mitochondria Isolated From Lungs and Mitochondria in Intact Lungs

The metabolic environment in isolated mitochondrial experiments is different from that in isolated perfused lung experiments. For instance, the environment in isolated mitochondria experiments is quite stable since the buffer volume is relatively large as compared to mitochondrial volume (ratio of mitochondrial volume to buffer volume is ~1/1000) (Wu et al., 2007; Zhang et al., 2018). On the other hand, lung bioenergetics are more sensitive to metabolic control in intact rat lungs because of the relatively large mitochondria/cytosol volume ratio (mitochondria to cytosol volume ratio of ~1/50) (Gan et al., 2011).

**Figure 11:**
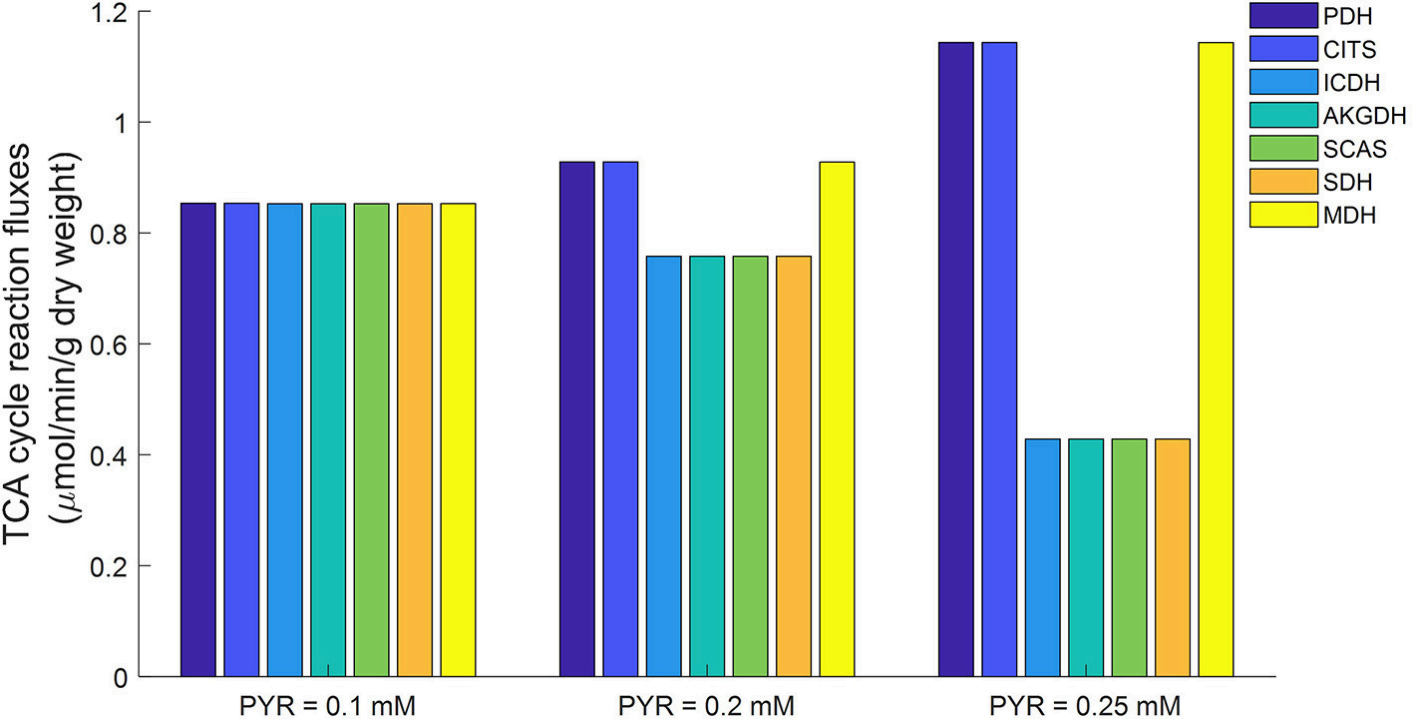
Model predicted mitochondrial TCA cycle fluxes as functions of cytosolic pyruvate (PYR) concentration using parameters in Table 2. When cytosolic PYR concentration is clamped below 0.1 mM, TCA cycle is seen to be functionally complete (equal fluxes at steady state). As cytosolic concentration increases, TCA cycle becomes increasingly functionally incomplete. PDH, Pyruvate dehydrogenase; CITS, Citrate synthase; ICDH, Isocitrate dehydrogenase; AKGDH, α-ketoglutarate dehydrogenase; SCAS, Succinyl-coenzyme A synthetase; SDH, Succinate dehydrogenase; MDH, Malate dehydrogenase.

Mitochondrial respiratory substrates can inhibit glycolytic rate via increased citrate production from mitochondria (Fisher and Dodia, 1984). For this study, conditions of abundant mitochondrial respiratory substrates were simulated using the lung tissue bioenergetics model by elevating cytosolic pyruvate concentration. The simulations in Figure 11 show that the fluxes of three TCA cycle reactions (namely PDH, CITS, and MDH) increased with increased cytosolic pyruvate concentration. The fluxes of the other TCA cycle reactions actually decreased with increased cytosolic pyruvate concentration. As a result, excessive mitochondrial citrate produced by the TCA cycle is released to the cytosol, which in turn inhibits glycolysis.

Previously we showed that the TCA cycle in mitochondria isolated from rat lung tissue is functionally incomplete (Zhang et al., 2018), with only half of the TCA cycle reactions (PDH, CITS, MDH) active while the other reactions are apparently inactive. However, as Figure 11 shows, a different TCA cycle behavior was observed in simulations generated using the lung tissue bioenergetics model. For such experimental conditions, the TCA cycle is complete and all the reactions are running at approximately equal rates. This difference in TCA cycle activity is potentially due to differences between the mitochondrial environment of the isolated mitochondria preparation and the isolated perfused lung preparation. For instance, the physiological pyruvate concentration in isolated perfused rat lung is around ~0.1 mM under normal conditions (Kadlecek et al., 2014), vs. a buffer pyruvate concentration of ~5 mM used in isolated mitochondrial experiments to ensure enough substrate availability for mitochondrial respiration (Fisher et al., 1973; Evans and Scholz, 1975; Fisher, 1975; Zhang et al., 2018).

Simulations using the proposed integrated lung tissue bioenergetics model provide a potential explanation for why the TCA cycle is apparently incomplete in isolated mitochondrial studies, but is complete in isolated perfused rat lung experiments. A higher cytosolic pyruvate concentration, such as that used in isolated mitochondrial studies, results in higher NADH generation in mitochondria. Isocitrate dehydrogenase (ICDH) is known to be inhibited by NADH (Qi et al., 2008; Zhang et al., 2018), with the ICDH activity decreasing as mitochondrial NADH/NAD ratio increases. As such, increased amounts of citrate is released into the cytosol through the mitochondrial transporter tricarboxylate carrier (TCC) resulting in an incomplete TCA cycle.

Based on the calculated mitochondria volume (13.1 μL in Table 2), the calculated mitochondria protein mass is around 13.1 mg in intact rat lung. This value is close to the model-estimated value (16.4 mg). Based on our previous study [5], mitochondria yield from lung tissue is around 2–3 mg/rat lung. That suggests that 80–85% of rat lung mitochondria is lost/inactivated during the isolation process.

### Lactate Regulation of Glycolytic Rate

Experimental data showed that exogenous lactate has a regulatory effect on glycolytic rate (Figure 3D) (Wolfe et al., 1979; Fisher and Dodia, 1984). However, the underlying mechanism for such an effect is not well understood. Different hypotheses have been proposed (Fisher and Dodia, 1984), including through alternations in cellular redox state or cellular energy states. However, mitochondrial control of the TCA cycle may also be contributing to lactate's regulation of the glycolytic rate.

In the cytosol, pyruvate is always in equilibrium with lactate since the enzyme lactate dehydrogenase (LDH) activity is much higher than that of other glycolytic enzymes. Thus, increasing exogenous lactate concentration increases pyruvate concentration in cytosol, which in turn inhibits glycolysis. This is consistent with the data in Figure 3D, which show that exogenous lactate has an inhibitory effect on rat lung glycolytic rates (Wolfe et al., 1979; Fisher and Dodia, 1984;Wang et al., 2004).

Even though the glucose utilization rate of rat lung is similar to that of heart and brain (Fisher, 1984), isolated perfused rat lungs produce significantly more lactate than hearts (Fisher, 1984) and skeletal muscle (Li et al., 2009). Forty to fifty percent of glucose consumed by lungs is released as lactate (Fisher, 1984). Thus, a significant fraction of NADH produced in the glycolytic pathway is consumed by lactate dehydrogenase. Only ~10% of NADH produced by glycolysis is transported into mitochondria by MA-shuttle (Fisher, 1984). Previous modeling studies have suggested that the MA-shuttle may have an important regulatory role on the bioenergetics of skeletal muscle and heart (Wu et al., 2007; Li et al., 2009). However, simulations using the proposed integrated lung bioenergetics models suggest that the role of the MA-shuttle may be less important in the intact rat lung.

### Model Limitations

As shown in Figures 3B,C, model-simulated endogenous lactate underestimates experimentally-measured lactate production (Kerr et al., 1979) when perfusate glucose concentration is low (0–0.5 mM). The model predicts that when exogenous glucose is low, lung mitochondria divert more pyruvate from endogenous substrates (e.g., alanine), thus resulting in lower endogenous lactate production. However, Figure 3C shows that experimentally measured endogenous lactate production (Kerr et al., 1979) is independent of perfusate glucose concentration. One possible reason for this could be that experimentally measured endogenous lactate production (Figure 3C) (Kerr et al., 1979) is overestimated since pyruvate and lactate were extracted using ion-exchange chromatography (Bassett and Fisher, 1976a; Kerr et al., 1979). This method cannot differentiate between lactate and other small anions potentially present in the perfusate (Bassett and Fisher, 1976a). It is likely that the production rate of those small anions is independent of perfusate glucose concentration.

Another possible reason for the inability of the model to account for the rate of lactate production under conditions of low perfusate glucose concentration could be that the activity of the enzyme alanine aminotransferase is dependent on ATP level. The rationale is that when glucose is low, protein degradation is stimulated in response to decreased ATP, resulting in more pyruvate formation from alanine. However, experimental data and model simulations in Figure 8 show that pyruvate production is decreased in the presence of rotenone (complex I inhibitor) which is known to decrease mitochondrial ATP (Bongard et al., 2013).

Glucose and alanine are considered major metabolic substrates for this rat lung bioenergetics model. Other substrates such as fatty acids and glycogen are not accounted for. Under normal conditions, fatty acid production accounts for < 5% of glucose consumption in isolated perfused rat lungs (Fisher, 1984). Thus, the contribution of fatty acid is considered insignificant. However, under low energy conditions, fatty acid could become a significant alternative energy source. For example, fatty acid oxidation may be activated by AMPK (AMP-activated protein kinase) when glucose is low (Connolly et al., 2016). Fatty acids were not accounted for in the present model due to the scarcity of experimental data regarding fatty acid as a source of energy in intact lungs.

Another limitation of the lung model is that it does not account for all ATP consumption processes, including consumption by active transporters, due to the scarcity of experimental data (Fisher et al., 1976; Kerr et al., 1979; Fisher and Dodia, 1981, 1984; Fisher, 1984; Kallet and Matthay, 2013; Zhang et al., 2018). Instead, the ATP consumption rate is modeled as a passive process whose rate is only dependent on the concentrations of ATP, ADP, and inorganic phosphate. Accounting for all ATP consumption processes and their regulatory effects could improve the ability of the model to simulate experimental data under conditions of low glucose concentration.

Another limitation of the lung model is that it does not account for metal ions, including calcium (Ca^2+^). Accounting for Ca^2+^ and other ions in the model would require adding transporters and buffering mechanisms for those ions, and accounting for their interactions with other metabolites in the different regions of the model. This would require experimental data about those processes from lungs and lung mitochondria to estimate the relevant model parameters descriptive of those processes. Such data are not currently available, and collecting such data is beyond the scope of this study.

## Conclusion

We have developed and validated an integrated mechanistic computational model of intact rat lung tissue bioenergetics by extending our recently developed integrated thermodynamically-constrained computational model of the bioenergetics of mitochondria isolated from rat lungs. The model was used to gain insights on how lung tissue glycolytic rate is regulated by exogenous substrates, such as glucose and lactate, and to assess differences in the bioenergetics of mitochondria isolated from lung tissue and those of mitochondria in intact lungs. To the best of our knowledge, this is the first model for intact lung tissue bioenergetics. The model provides a mechanistic and quantitative framework for integrating available lung bioenergetics data, and for testing novel hypotheses regarding the role of different cytosolic and mitochondrial processes in lung tissue bioenergetics under physiological and pathophysiological conditions. The MATLAB model code is available at the model sharing website (*physiome.org*) or upon request submitted to the corresponding author.

## Data Availability

The raw data supporting the conclusions of this manuscript will be made available by the authors, without undue reservation, to any qualified researcher.

## Author Contributions

XZ, RD, SA, DX, and AC: developed and validated the model. SA, XZ, RD, and EJ: choice and interpretation of experimental data. XZ, RD, SA, and AC: analyzed the data. SA, RD, and EJ: contributed funding and analysis tools. XZ, SA, and RD: wrote the paper. All authors approved the final manuscript before submission.

### Conflict of Interest Statement

The authors declare that the research was conducted in the absence of any commercial or financial relationships that could be construed as a potential conflict of interest.
