# Supplementary material for: Integrated Computational Model of Lung Tissue Bioenergetics
Source: Front Physiol. 2019 Mar 8;10:191. doi: 10.3389/fphys.2019.00191 (PMC6418344; doi:10.3389/fphys.2019.00191)
Supplement: Supplementary file 1 [file Data_Sheet_1.docx]

## Supporting Information

**“Integrated Computational Model of Lung Tissue Bioenergetics”**

**Xiao Zhang^1^, ^#^Ranjan K. Dash^1,4,5^, Anne V. Clough^2,6^, Dexuan Xie^7^, Elizabeth R. Jacobs^2,3^, ^#^Said H. Audi^1,2,3,4^**

^1^Department of Biomedical Engineering, Marquette University; ^2^Zablocki V.A. Medical Center; ^3^Division of Pulmonary and Critical Care Medicine, Medical College of Wisconsin; ^4^Department of Biomedical Engineering, Medical College of Wisconsin; ^5^Department of Physiology, Medical College of Wisconsin; ^6^Department of Mathematics, Statistics, and Computer Science, Marquette University; ^7^Department of Mathematical Sciences, University of Wisconsin-Milwaukee.

**Introduction:**

This supporting information file consists of three parts. *Part A* lists the reaction and transport processes in the integrated computational model of the rat lung tissue bioenergetics. *Part B* provides a lists the flux expressions for specific metabolic reaction and transport processes. *Part C* lists the model’s governing mass balance equations.

## Part A: Reaction and transport processes.

| Terms | Description |
| --- | --- |
| Subscript r | Reservoir region |
| Subscript b | Lung vascular (blood) region |
| Subscript c | Cytosolic region |
| Subscript m | Mitochondria matrix region |
| Subscript i | Inter-membrane space (IMS) |
| Species | Description |
| ADP | Adenosine diphosphate |
| ATP | Adenosine triphosphate |
| ALA | Alanine |
| ACoA | Acetyl-coenzyme A |
| ASP | Aspartate |
| AKG | α-Ketoglutarate (2-Oxoglutarate) |
| BPG | 1,3-Bisphosphoglyceric acid |
| CoA | Coenzyme A |
| CIT | Citrate |
| CytCr | Reduced cytochrome c |
| CytCo | Oxidized cytochrome c |
| DHAP | Dihydroxyacetone phosphate |
| F16BP | Fructose-1,6-bisphosphate |
| F6P | Fructose-6-phosphate |
| FUM | Fumarate |
| GAP | Glyceraldehyde-3-Phosphate |

**Table A1: Glossary**

| GDP | Guanidine diphosphate |
| --- | --- |
| G6P | Glucose-6-phosphate |
| GTP | Guanidine triphosphate |
| GLC | Glucose |
| GLU | Glutamate |
| MAL | Malate |
| OXA | Oxaloacetate |
| PYR | Pyruvate |
| PG6 | 6-phosphogluconolactone |
| PEP | Phosphoenolpyruvic acid |
| Pi | Inorganic phosphate |
| SUC | Succinate |
| SCoA | Succinyl-coenzyme A |
| UQH_2_ | Reduced ubiquinone |
| UQ | Oxidized ubiquinone |
| Cytosolic Reactions | Description |
| AA | Alanine Aminotransferase |
| AK | Adenylate kinase |
| ALD | Lumped aldolase and triose phosphate isomerase |
| HK | Hexokinase |
| GAPDH | Glyceraldehyde-3-phosphate dehydrogenase |
| G6PDH | Glucose-6-phosphate dehydrogenase |
| GR | Glutathione reductase |
| GPx | Glutathione peroxidase |
| LDH | Lactate dehydrogenase |
| PFK | Phosphofructokinase |
| PGI | Phosphoglucose isomerase |
| PGK | Phosphoglycerate kinase |
| PK | Pyruvate Kinase |
| 6PGDH | 6-Phosphogluconate dehydrogenase |

| Mitochondrial Reactions | Description |
| --- | --- |
| AKGDH | α-Ketoglutarate dehydrogenase |
| CITS | Citrate synthase |
| ICDH | Lumped aconitase and isocitrate dehydrogenase |
| CI | Complex I |
| CII | Complex II |
| CIII | Complex III |
| CIV | Complex IV |
| CV | Complex V |
| FH | Fumarate hydratase |
| GOT | Glutamate oxaloacetate (or Aspartate aminotransferase) |
| MDH | Malate dehydrogenase |
| NDK | Nucleoside diphosphokinase |
| PDH | Pyruvate dehydrogenase |
| SCAS | Succinyl-coenzyme A synthetase |
| SDH | Succinate dehydrogenase |
| Transporters | Description |
| ANT | Mitochondrial adenine nucleotide translocase |
| DCC | Mitochondrial dicarboxylate carrier |
| GAE | Mitochondrial glutamate-aspartate exchanger |
| GLUH | Mitochondrial glutamate-hydrogen cotransporter |
| GLUT | Glucose transporter between blood and cytosol |
| LACT | Lactate transporter between blood and cytosol |
| LEAK | Mitochondrial passive proton leak |
| OME | Mitochondrial α-Ketoglutarate (2-Oxoglutarate) malate exchanger |
| PYRT | Pyruvate transporter between blood and cytosol |
| PIC | Mitochondrial inorganic phosphate carrier |
| PIT | Inorganic phosphate-Na cotransporter between blood and cytosol |
| PYRH | Mitochondrial pyruvate-hydrogen cotransporter |
| TCC | Mitochondrial tricarboxylate carrier |
| MAS | Malate-Aspartate shuttle |

***Table A2: Reactions in the isolated perfused rat lung model***

| Reaction number | Enzyme | Reference reactions |
| --- | --- | --- |
| 1 | HK | GLC_c_+ATP_c_ ⇌ G6P_c_+ADP_c_+H_c_ |
| 2 | PGI | G6P_c_ ⇌ F6P_c_ |
| 3 | PFK | F6P_c_+ATP_c_ ⇌ F16BP+ADP_c_+H+ |
| 4 | ALD | F16BP_c_ ⇌ 2GAP_c_ |
| 5 | GAPDH | GAP_c_+Pi_c_+NAD_c_ ⇌ BPG+NADH_c_+H_c_ |
| 6 | PGK | BPG_c_ + ADP_c_ ⇌ PEP_c_+ATP_c_ |
| 7 | PK | PEP_c_+ADP_c_+H_c_ ⇌ PYR_c_+ATP_c_ |
| 8 | LDH | PYR_c_ + NADH_c_ + H_c_ ⇌ LAC_c_ + NAD_c_^+^ |
| 9 | G6PDH | G6P_c_ + NADP_c_ ⇌ PG6_c_ + NADPH_c_ + H_c_ |
| 10 | 6PGD | PG6_c_ + NADP_c_ ⇌ R5P_c_ + NADPH_c_ + H_c_ + CO_2_ |
| 11 | GR | GSSG_c_ + H_c_ + NADPH ⇌ 2GSH_c_ + NADP_c_+ |
| 12 | GPx | 2GSH_c_ + H_2_O_2c_ ⇌ GSSG_c_ + 2H_2_O |
| 13 | ATPase | ATP_c_ ⇌ ADP_c_ + Pi_c_+ H_c_ |
| 14 | AK | ATP_c_+ AMP_c_ ⇌ 2ADP_c_ |
| 15 | AA | PYR_c_ ⇌ ALA_c_ |
| 16 | PDH | PYR_m_+CoA_m_+NAD_m_ ⇌ ACoA_m_+CO_2_+NADH_m_+H_m_ |
| 17 | CITS | ACoA_m_+OXA_m_ ⇌ CIT_m_+CoA_m_+ 2H_m_ |
| 18 | ICDH | CIT_m_+NAD_m_ ⇌ AKG_m_+NADH_m_+CO_2_ |
| 19 | AKGDH | AKG_m_+CoA_m_+NAD_m_ ⇌ SCoA_m_+NADH_m_+CO_2_ |
| 20 | SCAS | SCoA_m_+GDP_m_+Pi_m_ ⇌ SUC_m_+GTP_m_+CoA_m_+H_m_ |
| 21 | NDK | GTP_m_+ADP_m_ ⇌ GDP_m_+ATP_m_ |
| 22 | FUM | FUM_m_ = MAL_m_ |
| 23 | MDH | MAL_m_+NAD_m_ ⇌ OXA_m_+NADH_m_+ H_m_ |
| 24 | GOT | ASP_m_+AKG_m_ ⇌ GLU_m_+OXA_m_ |
| 25 | CI | NADH_m_+UQ_m_+H_m_ ⇌ NAD_m_+UQH_2m_+ 4∆H |
| 26 | CII/SDH | SUC_m_+ UQ_m_ ⇌ FUM_m_+ UQH_2m_ |
| 27 | CIII | UQH_2,m_+2CytCo+2H_m_ ⇌ UQ_m_+2CytCr+ 4∆H |
| 28 | CIV | 2CytC_r_+0.5O_2_+2H_m_ ⇌ 2CytCo+H_2_O+2∆H |
| 29 | CV | ADP_m_+Pi_m_+H_m_+3∆H ⇌ ATP_m_ |

***Table A3: Transports in the isolated perfused rat lung model***

| Transport number | Enzyme | Reference reactions |
| --- | --- | --- |
| 1 | GLUT | GLC_b_ ⇌ GLC_c_ |
| 2 | PYRT | PYR_b_ + H_b_ ⇌ PYR_c_ + H_c_ |
| 3 | LACT | LAC_b_ + H_b_ ⇌ LAC_c_ + H_c_ |
| 4 | PIT | Pi_b_ ⇌ Pi_c_ |
| 5 | DCC (SUC) | SUC_m_ +Pi_c_ ⇌ SUC_c_+Pi_m_ |
| 6 | DCC (MAL) | MAL_m_+Pi_c_ ⇌ MAL_c_+Pi_m_ |
| 7 | TCC | MAL_c_+ CIT_m_ ⇌ MAL_m_+ CIT_c_ |
| 8 | PYRH | PYR_c_ + H_c_ ⇌ PYR_m_ + H_m_ |
| 9 | PIC | Pi_c_ + H_c_ ⇌ Pi_m_ + H_m_ |
| 10 | ANT | ADP_c_ + ATP_m_ ⇌ ADP_m_ + ATP_c_ |
| 11 | GLUH | GLU_c_+ H_c_ ⇌ GLU_m_+ H_m_ |
| 12 | MAS | NADH_c_ + NAD_m_ ⇌ NADH_m_ + NAD_c_ |
| 13 | LEAK | H_i_ ⇌ H_m_ |

## Part B: Flux expressions for specific transport process and metabolic reactions.

### *Cytosolic region*

### Reaction 1: Hexokinase (HK)

### GLC_c_ + ATP_c_ ⇌ G6P_c_+ ADP_c_ + H_c_

For this reaction, the reactant concentrations are denoted as A = $[GLC_{c}]$, B =$[ATP_{c}]$, C = $[G6P_{c}]$, D = $[ADP_{c}]$, and ATP/ADP is considered a co-factor pair. In addition, this enzyme is known to be inhibited by its product, G6P (Berg, 2002;Li et al., 2009). The flux equation is modified based on (Li et al., 2009) to account for the inhibitor effect. The overall reaction flux is given by

$J_{HK}=\frac{\frac{V_{maxf}}{K_{A}K_{B}}\left(\frac{K_{iC}}{K_{iC}+C}\right)\left(AB-\frac{CD}{K_{eq}^{'}}\right)}{\left(1+\frac{A}{K_{A}}\right)\left(1+\frac{C}{K_{C}}\right)\left(1+\frac{B}{K_{B}}+\frac{D}{K_{D}}\right)}$ (A1)

***Table A4: HK model kinetic parameters***

| **Parameter** | **Definition** | **Value** | **Source** |
| --- | --- | --- | --- |
| *K_A_* | GLC apparent Michaelis constant | 1×10^-4^ M | (Bakker et al., 1997) |
| *K_B_* | ATP apparent Michaelis constant | 1.16×10^-4^ M | (Bakker et al., 1997) |
| *K_C_* | G6P apparent Michaelis constant | 1×10^-4^ M | Assumed the same as K_A_ |
| *K_D_* | ADP apparent Michaelis constant | 1.26×10^-4^ M | (Bakker et al., 1997) |
| *K_iC_* | G6P inhibition constant | 27.8×10^-3^ M | (Li et al., 2009) |
| $\text{Δ}_{r}G_{HK}^{'0}$ | Gibbs free energy of the reaction (pH=7) | -24.41 kJ/mol | (Vinnakota et al., 2006) |

### Reaction 2: Phosphoglucose isomerase (PGI)

### G6P_c_ ⇌ F6P_c_

For this reaction, the reactant concentrations are denoted as A =$[G6P_{c}]$, B =$[F6P_{c}]$. The overall reaction flux equation is given by

$J_{PGI}=\frac{\frac{V_{maxf}}{K_{A}}\left(A-\frac{B}{K_{eq}^{'}}\right)}{\left(1+\frac{A}{K_{A}}\right)\left(1+\frac{B}{K_{B}}\right)}$ (A2)

**Table A5*: PGI model kinetic parameters***

| **Parameter** | **Definition** | **Value** | **Source** |
| --- | --- | --- | --- |
| *K_A_* | G6P apparent Michaelis constant | $0.59\times10^{-3} M$ | (Gaitonde et al., 1989) |
| *K_B_* | F6P apparent Michaelis constant | $0.095\times10^{-3} M$ | (Gaitonde et al., 1989) |
| $\text{Δ}_{r}G_{PGI}^{'0}$ | Gibbs free energy of the reaction (pH=7.4) | 3.18 kJ/mol | (Vinnakota et al., 2006) |

### Reaction 3: Phosphofructokinase (PFK)

### F6P_c_ + ATP_c_ ⇌ F16BP_c_+ADP_c_+H^+^

The reactant concentrations are denoted as A = $[F6P_{c}]$, B = $[ATP_{c}]$, C = $[F16BP_{c}]$, D = $[ADP_{c}]$.

PFK is the key regulatory enzyme for glycolysis. It follows sigmodial kinetics with respect to F6P. ATP allosterically inhibits PFK by decreasing its affinity for F6P (i.e. increasing apparent Michaelis constant of F6P), whereas AMP and ADP compete for the allosteric site and reverse the inhibitory action of ATP (Berg, 2002). In addition, PFK is inhibited by cytosolic citrate (Berg, 2002;Li et al., 2009).

Thus, the reaction flux equation is modified to account for the regulatory effects of ATP, ADP, AMP and citrate based on (Mulquiney and Kuchel, 1999). The proportion of PFK in active state (R state) is given by

$p=\frac{1}{1+L}$ (A3)

where

$L=\frac{\left(1+\frac{\left[ATP_{c}\right]}{K_{i,ATP}}\right)^{n}\left(1+\frac{\left[CIT_{c}\right]}{K_{i,CIT}}\right)^{n}}{\left(1+\frac{\left[F6P_{c}\right]}{K_{A}}+\frac{\left[F16BP\right]_{c}}{K_{C}}\right)^{n}\left(1+\frac{\left[AMP_{c}\right]}{K_{a,AMP}}\right)^{n}\left(1+\frac{\left[ADP_{c}\right]}{K_{a,ADP}}\right)^{n}}$ (A4)

Even though fructose 2,6-bisphosphate is also an activator of PFK, its physiological concentration (30 µM) is sufficient to fully activate PFK (half maximal activation occurs at ~2µM) (Heesbeen et al., 1989). Thus, its effect is not considered in this model.

The overall reaction flux equation $J_{PFK}$is given by

$J_{PFK}=\frac{p\frac{V_{maxf}}{K_{A}K_{B}}\left(AB-\frac{CD}{K_{eq}^{'}}\right)}{\left(1+\frac{A}{K_{A}}\right)\left(1+\frac{C}{K_{C}}\right)\left(1+\frac{B}{K_{B}}+\frac{D}{K_{D}}\right)}$ (A5)

The activation constants or inhibition constants were estimated based on available data from rat lung PFK shown in Figure A1. In Figure A1, symbols are experimental data obtained from *(Heesbeen et al., 1989)* and *(Abuelgassim et al., 1992)* and solid lines are model flux Equation A5 fits to the data

###

***Figure A1:* (A)** Effect of citrate concentration on normalized enzyme activities measured in isolated adult rat lung. Symbols are experimental data obtained from (Abuelgassim et al., 1992) and solid lines are model fits of Equation A5 to the data. **(B)** Effect of ATP concentration on apparent S0.5 of F6P measured in rat lung. S0.5 is defined as the concentration by which half maximal velocity is achieved. **(C)** Effect of AMP and ADP inhibition on normalized enzyme activity. Symbols in (B) and (C) are experimental data obtained from (Heesbeen et al., 1989) and solid lines are model fits. **(D)** Predicted cooperative feature of PFK with respect to F6P under different ATP concentrations.

***Table A6: PFK model kinetic parameters***

| **Parameter** | **Definition** | **Value** | **Source** |
| --- | --- | --- | --- |
| *K_A_* | F6P apparent Michaelis constant | $0.032\times10^{-3} M$ | (Campos et al., 1984) |
| *K_B_* | ATP apparent Michaelis constant | $0.020\times10^{-3} M$ | (Campos et al., 1984) |
| *K_C_* | F16BP apparent Michaelis constant | $0.032\times10^{-3} M$ | Assumed the same as K_A_ |
| *K_D_* | ADP apparent Michaelis constant | $0.020\times10^{-3} M$ | Assumed the same as K_B_ |
| *K_iCIT_* | CIT inhibition constant | $5.5\times10^{-3} M$ | Estimated from Figure A1 |
| *K_iATP_* | ATP inhibition constant | $4.9\times10^{-6} M$ | Estimated from Figure A1 |
| *K_aAMP_* | AMP activation constant | $1.75\times10^{-6} M$ | Estimated from Figure A1 |
| *K_aADP_* | ADP activation constant | $3.5\times10^{-6} M$ | Estimated from Figure A1 |
| *n* | Hill coefficient | 2.3 | Estimated from Figure A1 |
| $\text{Δ}_{r}G_{PFK}^{'0}$ | Gibbs free energy of the reaction (pH=7) | -23.24 kJ/mol | (Vinnakota et al., 2006) |

### Reaction 4: Aldolase and triose phosphate isomerase (ALD)

F16BP_c_ ⇌ 2GAP_c_

This is a lumped reaction of F16BP aldolase and triose phosphate isomerase

**F16BP Aldolase:** F16BP ⇌ GAP + DHAP

**Triose phosphate isomerase:** DHAP ⇌ GAP

The reactant concentrations are denoted as A = $[F16BP_{c}]$, B = $[GAP_{c}]$. The overall reaction flux equation $J_{ALD}$is given by

$J_{ALD}=\frac{\frac{V_{maxf}}{K_{A}}\left(A-\frac{B^{2}}{K_{eq}^{'}}\right)}{\left(1+\frac{A}{K_{A}}\right)\left(1+\frac{B^{2}}{K_{B}^{2}}\right)}$ (A6)

***Table A7: ALD model kinetic parameters***

| **Parameter** | **Definition** | **Value** | **Source** |
| --- | --- | --- | --- |
| *K_A_* | F16BP apparent Michaelis constant | $1.7\times10^{-6} M$ | (Midelfort et al., 1976) |
| *K_B_* | GAP apparent Michaelis constant | $1.7\times10^{-6} M$ | Assumed the same as *K_A_* |
| $\text{Δ}_{r}G_{ALD}^{'0}$ | Gibbs free energy of the reaction (pH=7) | 30.7 kJ/mol | (Vinnakota et al., 2006) |

Reaction 5: **Glyceraldehyde 3-Phosphate Dehydrogenase (GAPDH)**

### GAP_c_ + Pi_c_ + NAD_c_ ⇌ BPG_c_ + NADH_c_ + H_c_

The reactant concentrations are denoted as A = $[GAP_{c}]$, B = $[Pi_{c}]$, C =$[NAD_{c}]$, D = $[BPG_{c}]$, E =$[NADH_{c}]$. NAD/NADH are considered cofactor pairs. The overall reaction flux equation $J_{GAPDH}$is given by

$J_{GAPDH}=\frac{\frac{V_{maxf}}{K_{A}K_{B}}\left(ABC-\frac{DE}{K_{eq}^{'}}\right)}{\left(1+\frac{A}{K_{A}}\right)\left(1+\frac{C}{K_{C}}+\frac{E}{K_{E}}\right)\left(1+\frac{B}{K_{B}}\right)\left(1+\frac{D}{K_{D}}\right)}$ (A7)

***Table A8: GAPDH model kinetic parameters***

| **Parameter** | **Definition** | **Value** | **Source** |
| --- | --- | --- | --- |
| *K_A_* | GAP apparent Michaelis constant | $20.7\times10^{-6} M$ | (Mountassif et al., 2009) |
| *K_B_* | Pi apparent Michaelis constant | $2.9\times10^{-3} M$ | Set to initial concentration |
| *K_C_* | NAD apparent Michaelis constant | $17.8\times10^{-6} M$ | (Mountassif et al., 2009) |
| *K_D_* | BPG apparent Michaelis constant | $20.7\times10^{-6} M$ | Assumed the same as *K_A_* |
| *K_E_* | NADH apparent Michaelis constant | $17.8\times10^{-6} M$ | Assumed the same as *K_C_* |
| $\text{Δ}_{r}G_{GAPDH}^{'0}$ | Gibbs free energy of the reaction (pH=7) | -1.21 kJ/mol | (Vinnakota et al., 2006) |

### Reaction 6: Phosphoglycerate Kinase (PGK)

BPG_c_ + ADP_c_ ⇌ PEP_c_ + ATP_c_

The reactant concentrations are denoted as A = $[BPG_{c}]$, B = $[ADP_{c}]$, C =$[PEP_{c}]$, D = $[ATP_{c}]$. ADP/ATP is considered a cofactor pair. Thus, the overall reaction flux equation $J_{PGK}$is given by

$J_{PGK}=\frac{\frac{V_{maxf}}{K_{A}K_{B}}\left(AB-\frac{CD}{K_{eq}^{'}}\right)}{\left(1+\frac{A}{K_{A}}\right)\left(1+\frac{C}{K_{C}}\right)\left(1+\frac{B}{K_{B}}+\frac{D}{K_{D}}\right)}$ (A8)

***Table A9: PGK model kinetic parameters***

| **Parameter** | **Definition** | **Value** | **Source** |
| --- | --- | --- | --- |
| *K_A_* | BPG apparent Michaelis constant | $0.01\times10^{-3} M$ | (Sharma et al., 1980) |
| *K_B_* | ADP apparent Michaelis constant | $0.4\times10^{-3} M$ | Assumed the same as *K_D_* |
| *K_C_* | PEP apparent Michaelis constant | $0.01\times10^{-3} M$ | Assumed the same as *K_A_* |
| *K_D_* | ATP apparent Michaelis constant | $0.4\times10^{-3} M$ | (Sharma et al., 1980) |
| $\text{Δ}_{r}G_{PGK}^{'0}$ | Gibbs free energy of the reaction (pH=7) | -5.85 kJ/mol | (Vinnakota et al., 2006) |

### Reaction 7: Pyruvate Kinase (PK)

### PEP_c_ + ADP_c_ + H_c_ ⇌ PYR_c_ + ATP_c_

The reactant or regulator concentrations are denoted as A = $[PEP_{c}]$, B = $[ADP_{c}]$, C =$[PYR_{c}]$, D = $[ATP_{c}]$. E=$[F16BP_{c}]$.ADP/ATP is considered a cofactor pair. The enzyme is known to be strongly activated by low concentration of F16BP (Carbonell et al., 1973;Schering et al., 1982). The overall reaction flux equation $J_{PK}$is given by

$J_{PK}=\frac{\frac{V_{maxf}}{K_{A}K_{B}}\left(\frac{E}{K_{aE}+E}\right)\left(AB-\frac{CD}{K_{eq}^{'}}\right)}{\left(1+\frac{A}{K_{A}}\right)\left(1+\frac{C}{K_{C}}\right)\left(1+\frac{B}{K_{B}}+\frac{D}{K_{D}}\right)}$ (A9)

***Table A10: PK model kinetic parameters***

| **Parameter** | **Definition** | **Value** | **Source** |
| --- | --- | --- | --- |
| *K_A_* | PEP apparent Michaelis constant | $0.26\times10^{-3} M$ | (Schering et al., 1982) |
| *K_B_* | ADP apparent Michaelis constant | $0.40\times10^{-3} M$ | (Schering et al., 1982) |
| *K_C_* | PYR apparent Michaelis constant | $0.26\times10^{-3} M$ | Assume the same as *K_A_* |
| *K_D_* | ATP apparent Michaelis constant | $0.40\times10^{-3} M$ | Assume the same as *K_B_* |
| *K_aE_* | F16BP activation constant | $1\times10^{-6} M$ | (Schering et al., 1982) |
| $\text{Δ}_{r}G_{PK}^{'0}$ | Gibbs free energy of the reaction (pH=7) | -28.89 kJ/mol | (Vinnakota et al., 2006) |

### Reaction 8: Lactate Dehydrogenase (LDH)

PYR_c_ + NADH_c_ + H_c_ ⇌ LAC_c_ + NAD_c_^+^

The reactant concentrations are denoted as A = $[PYR_{c}]$, B = $[NADH_{c}]$, C =$[LAC_{c}]$, D = $[NAD_{c}]$. NAD/NADH is considered a cofactor pair. Thus, the overall reaction flux equation $J_{LDH}$is given by

$J_{LDH}=\frac{\frac{V_{maxf}}{K_{A}K_{B}}\left(AB-\frac{CD}{K_{eq}^{'}}\right)}{\left(1+\frac{A}{K_{A}}\right)\left(1+\frac{C}{K_{C}}\right)\left(1+\frac{B}{K_{B}}+\frac{D}{K_{D}}\right)}$ (A10)

***Table A11: LDH model kinetic parameters***

| **Parameter** | **Definition** | **Value** | **Source** |
| --- | --- | --- | --- |
| *K_A_* | PYR apparent Michaelis constant | $0.052\times10^{-3} M$ | (Wang et al., 2016) |
| *K_B_* | NADH apparent Michaelis constant | $0.68\times10^{-6} M$ | Fixed to initial concentration |
| *K_C_* | LAC apparent Michaelis constant | $4.934\times10^{-3} M$ | (Wang et al., 2016) |
| *K_D_* | NAD apparent Michaelis constant | $340\times10^{-6} M$ | Fixed to initial concentration |
| $\text{Δ}_{r}G_{LDH}^{'0}$ | Gibbs free energy of the reaction (pH=7) | -23.9 kJ/mol | (Vinnakota et al., 2006) |

### **Reactions 9 and 10: Pentose phosphate pathway reactions (PPP)**

*6-Phosphogluconate Dehydrogenase (6PGDH) (Li et al., 2011)*

G6P_c_ + 2NADP_c_ ⇌ R5P_c_ + 2NADPH_c_+ 2H_c_+ CO_2_; $\text{Δ}_{r}G^{'0}$ = -20.66 kJ/mol

### R5P_c_ ⇌ $\frac{5}{6}\text{G6P}_{c}$; $\text{Δ}_{r}G^{'0}$= -34.23 kJ/mol

***Overall equation for the pentose phosphate pathway:***

$\frac{1}{6}\text{G6P}_{c}\text{+2NADP}_{c}$⇌$\text{CO}_{2}\text{+2NADPH}_{c}\text{+2H}_{c}$*;* $\text{Δ}_{r}G^{'0}$= -54.89 kJ/mol

The reactant concentrations are denoted as A = $[G6P_{c}]$, B = $[NADP_{c}]$, C =$[CO_{2}]$, D = $[NADPH_{c}]$.

$J_{PPP}=\frac{\frac{V_{maxf}}{K_{A}^{1/6}K_{B}^{2}}\left(A^{1/6}B^{2}-\frac{CD^{2}}{K_{eq}^{'}}\right)}{\left(1+\frac{A^{1/6}}{K_{A}^{1/6}}+\frac{C}{K_{C}}\right)\left(1+\frac{B^{2}}{K_{B}^{2}}+\frac{D^{2}}{K_{D}^{2}}\right)}$ (A11)

***Table A12: PPP model kinetic parameters***

| **Parameter** | **Definition** | **Value** | **Source** |
| --- | --- | --- | --- |
| *K_A_* | G6P apparent Michaelis constant | $36\times10^{-6} M$ | (Hanau et al., 2010) |
| *K_B_* | NADP apparent Michaelis constant | $4.8\times10^{-6} M$ | (Hanau et al., 2010) |
| *K_C_* | PG6 apparent Michaelis constant | $36\times10^{-6} M$ | (Hanau et al., 2010) |
| *K_D_* | NADPH apparent Michaelis constant | $4.8\times10^{-6} M$ | (Hanau et al., 2010) |
| $\text{Δ}_{r}G_{PPP}^{'0}$ | Gibbs free energy of the reaction (pH=7) | -54.89 kJ/mol | (Li et al., 2011) |

### **Reaction 11: Glutathione reductase (GSR)**

GSSG_c_ + H_c_ + NADPH_c_ ⇌ 2GSH + NADP_c_^+^

$J_{GSR}=\frac{\frac{V_{maxf}}{K_{A}}\left(A-\frac{BC}{K_{eq}^{'}}\right)}{\left(1+\frac{A}{K_{A}}+\frac{B}{K_{B}}\right)\left(1+\frac{C}{K_{C}}\right)}$ (A12)

***Table A13: GSR model kinetic parameters***

| **Parameter** | **Definition** | **Value** | **Source** |
| --- | --- | --- | --- |
| *K_A_* | GSSG apparent Michaelis constant | $61\times10^{-6} M$ | (Mavis and Stellwagen, 1968) |
| *K_B_* | NADPH apparent Michaelis constant | $7.6\times10^{-6} M$ | (Mavis and Stellwagen, 1968) |
| *K_C_* | GSH apparent Michaelis constant | $61\times10^{-6} M$ | Assumed the same as *K_A_* |
| *K_D_* | NADP apparent Michaelis constant | $7.6\times10^{-6} M$ | Assumed the same as *K_B_* |
| $K_{eq,GSR}^{'0}$ | Equilibrium constant (pH=7) | 0.075 | (Bohme et al., 2000) |

### Reaction 12: GSH Peroxidase (GPx)

2GSH_c_ + H_2_O_2,c_ ⇌ GSSG_c_ + 2H_2_O

The reactant concentrations are denoted as A = $[GSH_{c}]$, B = $[H_{2}O_{2}]$, C =$[GSSG_{c}]$.The reactant H_2_O is ignored for this reaction. Thus, the overall reaction flux equation $J_{GPx}$is given by

$J_{GPx}=\frac{\frac{V_{maxf}}{K_{A}^{2}K_{B}}\left(A^{2}B-\frac{C}{K_{eq}^{'}}\right)}{\left(1+\frac{A^{2}}{K_{A}^{2}}+\frac{C}{K_{C}}\right)\left(1+\frac{B}{K_{B}}\right)}$ (A13)

***Table A14: GPx model kinetic parameters***

| **Parameter** | **Definition** | **Value** | **Source** |
| --- | --- | --- | --- |
| *K_A_* | GSH apparent Michaelis constant | $3\times10^{-3} M$ | (Little et al., 1970) |
| *K_B_* | H_2_O_2_ apparent Michaelis constant | $1\times10^{-6} M$ | (Little et al., 1970) |
| *K_C_* | GSSG apparent Michaelis constant | $3\times10^{-3} M$ | Assumed the same as *K_A_* |
| $\text{Δ}_{r}G_{GPx}^{'0}$ | Gibbs free energy of the reaction  (pH =7) | -275.7 kJ/mol | (Vinnakota et al., 2006) |

### Reaction 13: ATPase

### ATP_c_ ⇌ ADP_c_ + Pi_c_+ H_c_^+^

$J_{ATPase}=\frac{\frac{V_{maxf}}{K_{A}}\left(A-\frac{BC}{K_{eq}^{'}}\right)}{\left(1+\frac{A}{K_{A}}+\frac{B}{K_{B}}\right)\left(1+\frac{C}{K_{C}}\right)}$ (A14)

***Table A15: ATPase model kinetic parameters***

| **Parameter** | **Definition** | **Value** | **Source** |
| --- | --- | --- | --- |
| *K_A_* | ATP apparent Michaelis constant | $1\times10^{-3} M$ | Fixed |
| *K_B_* | ADP apparent Michaelis constant | $100\times10^{-6} M$ | Fixed |
| *K_C_* | Pi apparent Michaelis constant | $1\times10^{-3} M$ | Fixed |
| $\text{Δ}_{r}G_{ATPase}^{'0}$ | Gibbs free energy of the reaction (pH=7) | -36.03 kJ/mol | (Vinnakota et al., 2006) |

### Reaction 14: Adenylate kinase (AK)

AMP_c_ + ATP_c_ ⇌ 2ADP_c_

$J_{AK}=\frac{\frac{V_{maxf}}{K_{A}}\left(AB-\frac{C}{K_{eq}^{'}}\right)}{\left(1+\frac{A}{K_{A}}\right)\left(1+\frac{B}{K_{B}}\right)\left(1+\frac{C}{K_{C}}\right)^{2}}$ (A15)

***Table A16: AK model kinetic parameters***

| **Parameter** | **Definition** | **Value** | **Source** |
| --- | --- | --- | --- |
| *K_A_* | ATP apparent Michaelis constant | $1\times10^{-3} M$ | (Connolly et al., 2016) |
| *K_B_* | AMP apparent Michaelis constant | $10\times10^{-6} M$ | (Connolly et al., 2016) |
| *K_C_* | ADP apparent Michaelis constant | $100\times10^{-6} M$ | (Connolly et al., 2016) |
| $\text{Δ}_{r}G_{AK}^{'0}$ | Gibbs free energy of the reaction (pH=7) | -0.475 kJ/mol | (Vinnakota et al., 2006) |

For this rapidly equilibrating reaction,*V_maxf_* was set to be 1e7 nmol/min/lung.

### Reaction 15: Alanine Aminotransferase (AA) (Li et al., 2009)

ALA_c_ ⇌ PYR_c_

$J_{AA}=\frac{\frac{V_{maxf}}{K_{A}}\left(A-\frac{B}{K_{eq}^{'}}\right)}{\left(1+\frac{A}{K_{A}}\right)\left(1+\frac{B}{K_{B}}\right)}$ (A16)

***Table A17: AA model kinetic parameters***

| **Parameter** | **Definition** | **Value** | **Source** |
| --- | --- | --- | --- |
| *K_A_* | ALA apparent Michaelis constant | $1\times10^{-3} M$ | (Li et al., 2009) |
| *K_B_* | PYR apparent Michaelis constant | $100\times10^{-6} M$ | (Li et al., 2009) |
| $\text{Δ}_{r}G_{AA}^{'0}$ | Gibbs free energy of the reaction (pH=7) | -20 kJ/mol | (Li et al., 2009) |

### *Mitochondria region*

For the mitochondrial regions, the reaction flux equations are the same as those in our recently developed integrated model of the bioenergetics of mitochondria isolated from rat lungs (Zhang et al., 2018). Values of pertinent parameters were set to those estimated for our mitochondria model (Zhang et al., 2018). For completeness, the reaction flux equations and parameters are listed below.

### Reaction 16: Pyruvate dehydrogenase (PDH)

### PYR_m_ + CoA_m_ + NAD_m_ ⇌ ACoA_m_ + CO_2_ + NADH_m_ + H_m_^+^

For this reaction, the reactant concentrations are denoted as A = $[PYR_{m}]$, B = $[CoA_{m}]$, C = $[NAD_{m}]$, D = $[ACoA_{m}]$, E = $[CO_{2,m}]$, F = $[NADH_{m}]$. CO_2_ concentration is set at 1.32×10^-5^ M based on Henry’s law and is assumed to be constant. The participating co-factor pairs in the above reaction are NADH/NAD and CoA/ACoA. Thus, the overall reaction flux equation, $J_{PDH}$, is given by

$J_{PDH}=\frac{\frac{V_{maxf}}{K_{A}K_{B}K_{C}}\left(ABC-\frac{DEF}{K'_{eq}}\right)}{\left(1+\frac{A}{K_{A}}\right)\left(1+\frac{B}{K_{B}}+\frac{D}{K_{D}}\right)\left(1+\frac{C}{K_{C}}+\frac{F}{K_{F}}\right)\left(1+\frac{E}{K_{E}}\right)}$ (A17)

The above PDH-catalyzed reaction involves the generation of one proton. Thus, the pH-dependent apparent equilibrium constant for this reaction is defined as:

$K'_{eq}=e^{-\text{Δ}_{r}G_{PDH}^{'0}/RT}\times10^{pH_{m}-7}$ (A18)

***Table A18: PDH model kinetic parameters***

| **Parameter** | **Definition** | **Value** |
| --- | --- | --- |
| $K_{A}$ | PYR apparent Michaelis constant in isolated PDH | $2.52\times10^{-5} M$ |
| $K_{B}$ | CoA apparent Michaelis constant | $1.49\times10^{-5} M$ |
| $K_{C}$ | NAD apparent Michaelis constant | $3.50\times10^{-5} M$ |
| $K_{D}$ | ACoA apparent Michaelis constant | $1.49\times10^{-5} M$ |
| $K_{F}$ | NADH apparent Michaelis constant | $3.50\times10^{-5} M$ |
| $\text{Δ}_{r}G_{PDH}^{'0}$ | Gibbs free energy of the reaction (pH=7) | -38.64 kJ/mol |

### Reaction 17: Citrate synthase (CITS)

### ACoA_m_+OXA_m_ ⇌ CoA_m_+ CIT_m_ +2H_m_^+^

For this reaction, the reactant concentrations are denoted as A = $[ACoA_{m}]$, B = $[OXA_{m}]$, C = $[CoA_{m}]$, D = $[CIT_{m}]$. Since CoA and ACoA are co-factor pairs, the overall reaction flux equation,$J_{CITS}$, is given by,

$J_{CITS}=\frac{\frac{V_{maxf}}{K_{A}K_{B}}\left(AB-\frac{CD}{K'_{eq}}\right)}{\left(1+\frac{A}{K_{A}}+\frac{C}{K_{C}}\right)\left(1+\frac{B}{K_{B}}\right)\left(1+\frac{D}{K_{D}}\right)}$ (A19)

The reaction catalyzed by CITS generates two protons. Thus, the pH-dependent apparent equilibrium constant for this reaction is defined as:

$K'_{eq}=e^{-\text{Δ}_{r}G_{CITS}^{'0}/RT}\times10^{2pH_{m}-14}$ (A20)

***Table A19: CITS model kinetic parameters***

| **Parameter** | **Definition** | **Value** |
| --- | --- | --- |
| *K_A_* | ACoA apparent Michaelis constant | $3.90\times10^{-6} M$ |
| *K_B_* | OXA apparent Michaelis constant | $4.53\times10^{-6} M$ |
| *K_C_* | CoA apparent Michaelis constant | $57.9\times10^{-6} M$ |
| *K_D_* | CIT apparent Michaelis constant | $4.30\times10^{-3} M$ |
| $\text{Δ}_{r}G_{CITS}^{'0}$ | Gibbs free energy of the reaction (pH=7) | -36.61kJ/mol |

### Reaction 18: Aconitase and Iso-citrate dehydrogenase (ICDH)

The reactions catalyzed by aconitase and isocitrate (ISOCIT) dehydrogenase are:

***Aconitase:*** CIT ⇌ ISOCIT + H_2_O

***Isocitrate dehydrogenase:*** ISOCIT +NAD + H_2_O ⇌ AKG + NADH + CO_2_

Under the assumption that the reaction catalyzed by aconitase is rapidly equilibrating, the reactions catalyzed by aconitase and isocitrate can be lumped into the following reaction

CIT_m_ +NAD_m_ ⇌ AKG_m_ + NADH_m_ +CO_2_

For this reaction, the reactant concentrations are denoted as A = $[CIT_{m}]$, B = $[NAD_{m}]$, C = $[AKG_{m}]$, D = $[NADH_{m}]$, and E = $[CO_{2, m}]$, which is assumed to be constant.

$J_{ICDH}=\frac{\frac{V_{maxf}}{K_{A}K_{B}}\left(AB-\frac{CDE}{K'_{eq}}\right)}{\left(1+\frac{A}{K_{A}}\right)\left(1+\frac{C}{K_{C}}\right)\left(1+\frac{B}{K_{B}}+\frac{D}{K_{D}}\right)\left(1+\frac{E}{K_{E}}\right)}$ (A21)

The production of CO_2_ results in the generation of bicarbonate ions and one proton. Thus, the equilibrium constant for this reaction is defined as:

$K'_{eq}=e^{-\text{Δ}_{r}G_{ICDH}^{'0}/RT}10^{pH_{m}-7}$ (A22)

***Table A20: ICDH model kinetic parameters***

| **Parameter** | **Definition** | **Value** |
| --- | --- | --- |
| *K_A_* | CIT apparent Michaelis constant | $1.30\times10^{-3} M$ |
| *K_B_* | NAD apparent Michaelis constant | $500\times10^{-6} M$ |
| *K_C_* | AKG apparent Michaelis constant | $3.50\times10^{-6} M$ |
| *K_D_* | NADH apparent Michaelis constant | $4.70\times10^{-6} M$ |
| $\text{Δ}_{r}G_{ICDH}^{'0}$ | Gibbs free energy of the reaction (pH=7) | 2.81 KJ/mol |

### Reaction 19: AKG dehydrogenase (AKGDH)

### AKG_m_ + CoA_m_ + NAD_m_ ⇌ SCoA_m_+NADH_m_+CO_2_

For this reaction, the reactant concentrations are denoted as A =$[AKG_{m}]$, B = $[CoA_{m}]$, C = $[NAD_{m}]$, D = $[SCoA_{m}]$, E = $[NADH_{m}]$, and F = $[CO_{2}]$. The participating co-factor pairs in the above reaction are CoA/ACoA, and NADH/NAD. Thus, the overall reaction flux equation $J_{AKGDH}$ is given below.

$J_{AKGDH}=\frac{\frac{V_{maxf}}{K_{A}K_{B}K_{C}}\left(ABC-\frac{DEF}{K'_{eq}}\right)}{\left(1+\frac{C}{K_{C}}+\frac{E}{K_{E}}\right)\left(1+\frac{B}{K_{B}}+\frac{D}{K_{D}}\right)\left(1+\frac{A}{K_{A}}\right)}$ (A23)

Since the CO_2_ produced results in the generation of a bicarbonate ion and one proton, the equilibrium constant for this reaction is defined as

$K'_{eq}=e^{-\text{Δ}_{r}G_{AKGDH}^{'0}/RT}10^{pH_{m}-7}$ (A24)

***Table A21: AKGDH model kinetic parameters***

| **Parameter** | **Definition** | **Value** |
| --- | --- | --- |
| *K_A_* | AKG apparent Michaelis constant | $85.9\times10^{-6} M$ |
| *K_B_* | CoA apparent Michaelis constant | $1.63\times10^{-6} M$ |
| *K_C_* | NAD apparent Michaelis constant | $46.6\times10^{-6} M$ |
| *K_D_* | SCoA apparent Michaelis constant | $1.63\times10^{-6} M$ |
| *K_E_* | NADH apparent Michaelis constant | $7.14\times10^{-6} M$ |
| $\text{Δ}_{r}G_{AKGDH}^{'0}$ | Gibbs free energy of the reaction (pH=7) | -37.08 KJ/mol |

**Reaction 20: SCoA synthetase (SCAS)**

SCoA_m_ + GDP_m_ + Pi_m_ ⇌ SUC_m_ + GTP_m_ + CoA_m_ + H_m_^+^

For this reaction, the reactant concentrations are denoted as A = $[SCoA_{m}]$, B = $[GDP_{m}]$, C = $[Pi_{m}]$, D = $[SUC_{m}]$, E =$[GTP_{m}]$, and F = $[CoA_{m}]$. The participating co-factor pairs in this reaction are SCoA/CoA, ADP/ATP, ATP/Pi, and SCoA/SUC. Therefore, the overall reaction flux equation $J_{SCAS}$ is given by:

$J_{SCAS}=\frac{\frac{V_{maxf}}{K_{A}K_{B}K_{C}}\left(ABC-\frac{DEF}{K'_{eq}}\right)}{\left(1+\frac{A}{K_{A}}+\frac{D}{K_{D}}+\frac{F}{K_{F}}+\frac{DF}{K_{D}K_{F}}\right)\left(1+\frac{B}{K_{B}}+\frac{C}{K_{C}}+\frac{E}{K_{E}}+\frac{BC}{K_{B}K_{C}}\right)}$ (A25)

This reaction involves the generation of one proton, thus, the equilibrium constant for this reaction is defined as

$K'_{eq}=e^{-\text{Δ}_{r}G_{SCAS}^{'0}/RT}10^{pH_{m}-7}$ (A26)

***Table A22: SCAS model kinetic parameters***

| **Parameter** | **Definition** | **Value** |
| --- | --- | --- |
| *K_A_* | SCoA apparent Michaelis constant | $12.5\times10^{-6} M$ |
| *K_B_* | GDP apparent Michaelis constant | $5.70\times10^{-6} M$ |
| *K_C_* | Pi apparent Michaelis constant | $2.10\times10^{-3} M$ |
| *K_D_* | SUC apparent Michaelis constant | $451\times10^{-6} M$ |
| *K_E_* | GTP apparent Michaelis constant | $23.1\times10^{-6} M$ |
| *K_F_* | CoA apparent Michaelis constant | $17.7\times10^{-6} M$ |
| $\text{Δ}_{r}G_{SCAS}^{'0}$ | Gibbs free energy of the reaction (pH=7) | 1.26 KJ/mol |

**Reaction 21: Nucleoside diphosphokinase (NDK)**

GTP_m_+ADP_m_ ⇌ GDP_m_+ATP_m_

For this reaction, the reactant concentrations are denoted as A =$[GTP_{m}]$, B = $[ADP_{m}]$, C = $[GDP_{m}]$, and D = $[ATP_{m}]$, and the overall reaction flux equation is given by:

$J_{NDK}=\frac{\frac{V_{maxf}}{K_{A}K_{B}}\left(AB-\frac{CD}{K'_{eq}}\right)}{\left(1+\frac{A}{K_{A}}+\frac{C}{K_{C}}\right)\left(1+\frac{B}{K_{B}}+\frac{D}{K_{D}}\right)}$ (A27)

***Table A23: NDK model kinetic parameters***

| **Parameter** | **Definition** | **Value** | **Source** |
| --- | --- | --- | --- |
| *K_A_* | GTP apparent Michaelis constant | $111\times10^{-6}$ M | (Wu et al., 2007) |
| *K_B_* | ADP apparent Michaelis constant | $100\times10^{-6}$ M | (Wu et al., 2007) |
| *K_C_* | GDP apparent Michaelis constant | $260\times10^{-6}$ M | (Wu et al., 2007) |
| *K_D_* | ATP apparent Michaelis constant | $278\times10^{-6}$ M | (Wu et al., 2007) |
| $\text{Δ}_{r}G_{NDK}^{'0}$ | Gibbs free energy of the reaction (pH=7) | 0 KJ/mol | (Li et al., 2012) |

Parameters in the above reaction do not affect the simulations in the main paper since the reaction is assumed to be rapidly equilibrating.

**Reaction 22: Fumarate Hydratase (FH)**

FUM_m_ ⇌ MAL_m_

For this rapidly equilibrating reaction, the reactant concentrations are denoted as A = $[FUM_{m}]$, and B = $[MAL_{m}]$.

$J_{FH}=\frac{\frac{V_{maxf}}{K_{A}K_{B}}\left(A-\frac{B}{K'_{eq}}\right)}{\left(1+\frac{A}{K_{A}}\right)\left(1+\frac{B}{K_{B}}\right)}$ (A28)

***Table A24: FH model kinetic parameters***

| **Parameter** | **Definition** | **Value** |
| --- | --- | --- |
| *K_A_* | FUM apparent Michaelis constant | $1.8\times10^{-3} M$ |
| *K_B_* | MAL apparent Michaelis constant | $1.8\times10^{-3} M$ |
| $\text{Δ}_{r}G_{FH}^{'0}$ | Gibbs free energy of the reaction (pH=7) | -3.6 KJ/mol |

**Reaction 23: Malate Dehydrogenase (MDH)**

MAL_m_ + NAD_m_ ⇌ OXA_m_ + NADH_m_ + H_m_^+^

For this reaction, the reactant concentrations are denoted as A = $[NAD_{m}]$, B = $[MAL_{m}]$, C = $[OXA_{m}]$, D = $[NADH_{m}]$, the participating co-factor pairs are NADH/NAD, and MAL is known to be competitive inhibitor of OXA (Heyde and Ainsworth, 1968). Thus, the overall reaction flux equation is given by:

$J_{MDH}=\frac{\frac{V_{maxf}}{K_{A}K_{B}}\left(AB-\frac{CD}{K'_{eq}}\right)}{\left(1+\frac{B}{K_{B}}+\frac{C}{K_{C}}\right)\left(1+\frac{A}{K_{A}}+\frac{D}{K_{D}}\right)}$ (A29)

The above reaction involves the generation of one proton, thus, the equilibrium constant for this reaction is defined as

$K'_{eq}=e^{-\text{Δ}_{r}G_{MDH}^{'0}/RT}10^{pH_{m}-7}$ (A30)

***Table A25: MDH model kinetic parameters***

| **Parameter** | **Definition** | **Value** |
| --- | --- | --- |
| *K_A_* | MAL apparent Michaelis constant | $155\times10^{-6} M$ |
| *K_B_* | NAD apparent Michaelis constant | $1.10\times10^{-3} M$ |
| *K_C_* | OXA apparent Michaelis constant | $3.38\times10^{-6} M$ |
| *K_D_* | NADH apparent Michaelis constant | $36.1\times10^{-6} M$ |
| $\text{Δ}_{r}G_{MDH}^{'0}$ | Gibbs free energy of the reaction (pH=7) | 28.83 KJ/mol |

**Reaction 24: Glutamate oxaloacetate transaminase (GOT)**

ASP_m_ +AKG_m_ ⇌ GLU_m_ + OXA_m_

For this reaction, the reactant concentrations are denoted as A = $[ASP_{m}]$, B = $[AKG_{m}]$, C = $[GLU_{m}]$, D = $[OXA_{m}]$, and the overall reaction flux equation is given by:

$J_{GOT}=\frac{\frac{V_{maxf}}{K_{A}K_{B}}\left(AB-\frac{CD}{K'_{eq}}\right)}{\left(1+\frac{A}{K_{A}}\right)\left(1+\frac{B}{K_{B}}\right)\left(1+\frac{C}{K_{C}}\right)\left(1+\frac{D}{K_{D}}\right)}$ (A31)

The equilibrium constant for this reaction is:

$K'_{eq}=e^{-\text{Δ}_{r}G_{GOT}^{'0}/RT}$ (A32)

***Table A26: GOT model kinetic parameters***

| **Parameters** | **Definition** | **Value** |
| --- | --- | --- |
| *K_A_* | ASP apparent Michaelis constant | $3.9\times10^{-3} M$ |
| *K_B_* | ΑKG apparent Michaelis constant | $430\times10^{-6} M$ |
| *K_C_* | GLU apparent Michaelis constant | $8.9\times10^{-3} M$ |
| *K_D_* | OXA apparent Michaelis constant | $88\times10^{-6} M$ |
| $\text{Δ}_{r}G_{GOT}^{'0}$ | Gibbs free energy of the reaction (pH=7) | -1.31KJ/mol |

**Reaction 25: Complex I (CI)**

NADH_m_ + UQ_m_ + H_m_ ⇌ NAD_m_ + UQH_2m_ + 4ΔH

The above reaction, which is catalyzed by complex I, involves pumping four protons (ΔH) from the mitochondria matrix into the inter-membrane space. Thus, the reaction flux is dependent on proton motive force defined as $\Delta G_{H}=F\Delta\Psi_{m}+\left(RT\right)ln\left([H_{i}^{+}]/[H_{m}^{+}]\right)$, where *F* is the Faraday’s constant and $\Delta\Psi_{m}$ is the mitochondrial membrane potential. To take this dependency into account, the kinetic parameters was modified to be dependent on membrane potential (Dash and Beard, 2008;Dash et al., 2009).

For this reaction, the reactant concentrations are denoted as A = $[NADH_{m}]$, B = $[UQ_{m}]$, C = $[NAD_{m}]$, D = $[UQH_{2m}]$, and the participating co-factor pairs in this reaction are NAD/ NADH, and UQ/UQH_2_. Thus, the overall reaction flux equation is given by:

$J_{CI}=\frac{\frac{V_{maxf}}{K_{A}K_{B}}\left\{\begin{matrix} \exp\left(\frac{-\beta_{CI}(4\Delta G_{H}+\text{Δ}_{r}G_{CI}^{'0})}{RT}\right)AB \\ -\exp\left(\frac{-\left(\beta_{CI}-1\right)(4\Delta G_{H}+\text{Δ}_{r}G_{CI}^{'0})}{RT}\right)CD \end{matrix}\right\}}{\left(1+\frac{A}{K_{A}}+\frac{C}{K_{C}}\right)\left(1+\frac{B}{K_{B}}+\frac{D}{K_{D}}\right)}$ (A33)

***A27: CI model kinetic parameters***

| **Parameter** | **Definition** | **Value** |
| --- | --- | --- |
| *K_A_* | NADH apparent Michaelis constant | $1.50\times10^{-6} M$ |
| *K_B_* | UQ apparent Michaelis constant | $58.1\times10^{-6} M$ |
| *K_C_* | NAD apparent Michaelis constant | $428\times10^{-6} M$ |
| *K_D_* | UQH_2_ apparent Michaelis constant | $519\times10^{-6} M$ |
| *β_CI_* | Complex I free energy barrier | 0.5 |
| $\text{Δ}_{r}G_{CI}^{'0}$ | Gibbs free energy of the reaction (pH=7) | -69.37KJ/mol |

**Reaction 26: Complex II (CII)/succinate dehydrogenase (SDH)**

In this model, CII/SDH redox reaction is mathematically represented by the following reaction catalyzed by succinate dehydrogenase:

SUC_m_+ UQ_m_ ⇌ FUM_m_+ UQH_2m_

The reactant concentrations are denoted as A = $[SUC_{m}]$, B = $[UQ_{m}]$, C = $[FUM_{m}]$, and D = $[UQH_{2,m}]$. The overall reaction flux equation is given by:

$J_{SDH}=\frac{\frac{V_{maxf}}{K_{A}K_{B}}\left(AB-\frac{CD}{K'_{eq}}\right)}{\left(1+\frac{A}{K_{A}}\right)\left(1+\frac{C}{K_{C}}\right)\left(1+\frac{B}{K_{B}}+\frac{D}{K_{D}}\right)}$ (A34)

The equilibrium constant and thermodynamic constrains are properly accounted for, and have been validated in our recently published paper (Zhang et al., 2018) and in models developed by other groups (Cortassa et al., 2003;Cortassa et al., 2017).

***Table A28: SDH model kinetic parameters***

| **Parameter** | **Definition** | **Value** |
| --- | --- | --- |
| *K_A_* | SUC apparent Michaelis constant | $1.80\times10^{-3} M$ |
| *K_B_* | UQ apparent Michaelis constant | $58.1\times10^{-6} M$ |
| *K_C_* | FUM apparent Michaelis constant | $1.80\times10^{-3} M$ |
| *K_D_* | UQH_2_ apparent Michaelis constant | $519\times10^{-6} M$ |
| $\text{Δ}_{r}G_{SDH}^{'0}$ | Gibbs free energy of the reaction (pH=7) | -4.63 KJ/mol |

**Reaction 27: Complex III (CIII)**

UQH_2,m_+2CytCo+2H_m_ ⇌ UQ_m_+2CytCr+ 4∆H

For this reaction, the reactant concentrations are denoted as A = $[UQH_{2,m}]$, B = $[CytCo]$, C = $[UQ_{m}]$, D = $[CytCr]$, and the participating co-factor pairs are UQ_m_/UQH_2,m_, and CytC_r_/CytC_o_. In addition, the above reaction, catalyzed by complex III, involves pumping four protons from mitochondria matrix into inter-membrane space. Thus, the overall reaction flux equation is given by:

$J_{CIII}=\frac{\frac{V_{maxf}}{K_{A}K_{B}^{2}}\left(\begin{matrix} \exp\left\{\beta_{CIII}\left(\frac{-4\Delta G_{H}}{RT}+\frac{2F\Delta\Psi_{m}}{RT}-\frac{\text{Δ}_{r}G_{CIII}^{'0}}{RT}\right)\right\}AB^{2} \\ -\exp\left\{\left(\beta_{CIII}-1\right)\left(\frac{-4\Delta G_{H}}{RT}+\frac{2F\Delta\Psi_{m}}{RT}-\frac{\text{Δ}_{r}G_{CIII}^{'0}}{RT}\right)\right\}CD^{2} \end{matrix}\right)}{\left(1+\frac{A}{K_{A}}+\frac{C}{K_{C}}\right)\left(1+\frac{B^{2}}{K_{B}^{2}}+\frac{D^{2}}{K_{D}^{2}}\right)}$ (A35)

***Table A29: CIII model kinetic parameters***

| **Parameter** | **Definition** | **Value** |
| --- | --- | --- |
| *K_A_* | UQH_2_ apparent Michaelis constant | $4.66\times10^{-6} M$ |
| *K_B_* | CytCo apparent Michaelis constant | $3.76\times10^{-6} M$ |
| *K_C_* | UQ apparent Michaelis constant | $4.08\times10^{-6} M$ |
| *K_D_* | CytCr apparent Michaelis constant | $4.91\times10^{-6} M$ |
| *β_CIII_* | Complex III free energy barrier | 0.5 |
| $\text{Δ}_{r}G_{CIII}^{'0}$ | Gibbs free energy of the reaction (pH=7) | -32.53KJ/mol |

**Reaction 28: Complex IV (CIV)**

2CytCr + 0.5O_2_ + 2H_m_ ⇌ 2CytCo + H_2_O + 2ΔH

For this reaction, the reactant concentrations are denoted as A = $[CytCr]$, B = $[O_{2}]$, C =$[CytCo]$, and the participating co-factor pair is CytCr/CytCo. In addition, two protons are pumped from the matrix side to the inter-membrane space. Another two protons are consumed in the matrix side. The overall reaction flux equation is given by:

$J_{CIV}= \frac{\frac{V_{maxf}}{K_{A}^{2}K_{B}^{0.5}}\left(\begin{matrix} \exp\left\{\beta_{IV}\left(\frac{-2\Delta G_{H}}{RT}-\frac{2F\Delta\Psi_{m}}{RT}-\frac{\text{Δ}_{r}G_{CIV}^{'0}}{RT}\right)\right\}A^{2}B^{0.5} \\ -\exp\left\{\left(\beta_{IV}-1\right)\left(\frac{-2\Delta G_{H}}{RT}-\frac{2F\Delta\Psi_{m}}{RT}-\frac{\text{Δ}_{r}G_{CIV}^{'0}}{RT}\right)\right\}C^{2} \end{matrix}\right)}{\left(1+\frac{A^{2}}{K_{A}^{2}}+\frac{C^{2}}{K_{C}^{2}}\right)\left(1+\frac{B^{0.5}}{K_{B}^{0.5}}\right)}$ (A36)

***Table A30: CIV model kinetic parameters***

| **Parameters** | **Definition** | **Value** |
| --- | --- | --- |
| *K_A_* | CytCr apparent Michaelis constant | $680\times10^{-6} M$ |
| *K_B_* | Oxygen apparent Michaelis constant | $5.4\times10^{-6} M$ |
| *K_C_* | CytCo apparent Michaelis constant | $680\times10^{-6} M$ |
| *β_CIV_* | Complex IV free energy barrier | 0.5 |
| $\text{Δ}_{r}G_{CIV}^{'0}$ | Gibbs free energy of the reaction (pH=7) | -122.94KJ/mol |

**Reaction 29: Complex V (CV)**

ADP_m_ + Pi_m_ +3H_i_ + H_m_^+^ ⇌ ATP_m_ + 3H_m_^+^

For this reaction, the reactant concentrations are denoted as A = $[ADP_{m}]$, B = $[Pi_{m}]$, C = $[ATP_{m}]$, and the participating co-factor pair is ADP/ATP. Thus, the overall reaction flux equation is given by:

$J_{CV}=\frac{\frac{V_{maxf}}{K_{A}K_{B}}\left\{\begin{matrix} \exp\left(\frac{3\beta_{V}(\Delta G_{H}-\text{Δ}_{r}G_{CV}^{'0})}{RT}\right)AB \\ -\exp\left(\frac{3\left(\beta_{V}-1\right)(\Delta G_{H}-\text{Δ}_{r}G_{CV}^{'0})}{RT}\right)C \end{matrix}\right\}}{\left(1+\frac{A}{K_{A}}+\frac{C}{K_{C}}\right)\left(1+\frac{B}{K_{B}}\right)}$ (A37)

***Table A31: CV model kinetic parameters***

| **Parameter** | **Definition** | **Value** |
| --- | --- | --- |
| *K_A_* | ADP apparent Michaelis constant | $10\times10^{-6} M$ |
| *K_B_* | Pi apparent Michaelis constant | $0.5\times10^{-3} M$ |
| *K_C_* | ATP apparent Michaelis constant | $1\times10^{-3} M$ |
| $\beta_{CV}$ | Complex V free energy barrier | 0.5 |
| $\text{Δ}_{r}G_{CV}^{'0}$ | Gibbs free energy of the reaction (pH=7) | 36.03KJ/mol |

### Transport Fluxes

### *Transport 1: Glucose transporter (GLUT)*

Glucose transport between blood and cytosol is controlled by AMPK (AMP-activated protein kinase). As energy charge falls, glucose transport is activated by AMPK. Thus, the flux equation is modified to account for the cytosolic AMP effect on glucose transport.

GLC_b_ ⇌ GLC_c_

$J_{ GLUT}=\frac{T_{\max f}\left(\frac{AMP_{c}}{K_{a,AMP}+AMP_{c}}\right)\left(\frac{[GLC_{b}]}{K_{GLC}}-\frac{[GLC_{c}]}{K_{GLC}}\right)}{1+\frac{[GLC_{b}]}{K_{GLC}}+\frac{[GLC_{c}]}{K_{GLC}}}$ (A38)

***Table A32: GLUT model kinetic parameters***

| **Parameter** | **Definition** | **Value** | **Source** |
| --- | --- | --- | --- |
| *K_GLC_* | GLC apparent Michaelis constant | $3.4\times10^{-3} M$ | (Kerr et al., 1979) |
| *K_a, AMP_* | AMP activation constant | $0.21\times10^{-3} M$ | Fixed to AMP initial concentration |

### *Transport 2: Pyruvate transporter (PYRT)*

This transport process is catalyzed by the enzyme monocarboxylate transporter (MCT). *K_m_* for pyruvate and lactate are 0.1 mM and 0.7 mM, respectively (Johnson et al., 2011).

PYR_b_ + H_b_^+^ ⇌ PYR_c_ *+* H_c_^+^

$J_{ PYRT}=\frac{T_{\max f}\left(\frac{[PYR_{b}][H_{b}^{+}]}{K_{PYR}K_{H}}-\frac{[PYR_{c}][H_{c}^{+}]}{K_{PYR}K_{H}}\right)}{1+\frac{[PYR_{b}]}{K_{PYR}}+\frac{[PYR_{c}]}{K_{PYR}}+\frac{[H_{b}^{+}]}{K_{H}}+\frac{[H_{c}^{+}]}{K_{H}}+\frac{[PYR_{b}]}{K_{PYR}}\frac{[H_{b}^{+}]}{K_{H}}+\frac{[PYR_{c}]}{K_{PYR}}\frac{[H_{c}^{+}]}{K_{H}}}$ (A39)

***Table A33: PYRT model kinetic parameters***

| **Parameters** | **Definition** | **Value** | **Source** |
| --- | --- | --- | --- |
| *K_PYR_* | PYR apparent Michaelis constant | $0.1\times10^{-3} M$ | (Johnson et al., 2011) |

### *Transport 3: Lactate transporter (LACT)*

LAC_b_ + H_b_^+^ ⇌ LAC_c_ *+* H_c_^+^

$J_{ LACT}=\frac{T_{\max f}\left(\frac{[LAC_{b}][H_{b}^{+}]}{K_{LAC}K_{H}}-\frac{[LAC_{c}][H_{c}^{+}]}{K_{LAC}K_{H}}\right)}{1+\frac{[LAC_{b}]}{K_{LAC}}+\frac{[LAC_{c}]}{K_{LAC}}+\frac{[H_{b}^{+}]}{K_{H}}+\frac{[H_{c}^{+}]}{K_{H}}+\frac{[LAC_{b}]}{K_{LAC}}\frac{[H_{b}^{+}]}{K_{H}}+\frac{[LAC_{c}]}{K_{LAC}}\frac{[H_{c}^{+}]}{K_{H}}}$ (A40)

***Table A34: LACT model kinetic parameters***

| **Parameters** | **Definition** | **Value** | **Source** |
| --- | --- | --- | --- |
| *K_LAC_* | LAC apparent Michaelis constant | $0.7\times10^{-3} M$ | (Johnson et al., 2011) |

### *Transport 4: Phosphate transporter (PIT)*

Phosphate is co-transported with sodium ions (Na^+^) between blood and cytosol. Na^+^ concentration is assumed to be constant in this model. Therefore, the flux equation is simplified to

$J_{ PIT}=\frac{T_{\max f}\left(\frac{[Pi_{b}]}{K_{Pi}}-\frac{[Pi_{c}]}{K_{Pi}}\right)}{1+\frac{[Pi_{b}]}{K_{Pi}}+\frac{[Pi_{c}]}{K_{Pi}}}$ (A41)

***Table A35: PIT model kinetic parameters***

| **Parameters** | **Definition** | **Value** | **Source** |
| --- | --- | --- | --- |
| *K_Pi_* | Pi apparent Michaelis constant | $2\times10^{-3} M$ | Fixed |

### *Transport 5&6: Dicarboxylate Carrier (DCC)*

DCC (SUC): Pi_m_ + SUC_c_ ⇌ Pi_c_ + SUC_m_

DCC (MAL): Pi_m_ + MAL_c_ ⇌ Pi_c_ + MAL_m_

$J_{ DCC(MAL)}=\frac{T_{\max f}\left(\frac{\left[Pi_{m}\right]\left[MAL_{c}\right]}{K_{Pi}K_{MAL}}-\frac{\left[Pi_{c}\right]\left[MAL_{m}\right]}{K_{Pi}K_{MAL}}\right)}{\left(\begin{matrix} 1+\frac{\left[Pi_{m}\right]}{K_{Pi}}+\frac{\left[Pi_{c}\right]}{K_{Pi}}+\frac{\left[MAL_{m}\right]}{K_{MAL}}+\frac{\left[MAL_{c}\right]}{K_{MAL}}+\frac{\left[SUC_{m}\right]}{K_{SUC}}+\frac{\left[SUC_{c}\right]}{K_{SUC}}+ \\ \frac{\left[MAL_{c}\right]\left[Pi_{m}\right]}{K_{MAL}K_{Pi}}+\frac{\left[MAL_{m}\right]\left[Pi_{c}\right]}{K_{MAL}K_{Pi}}+\frac{\left[SUC_{c}\right]\left[Pi_{m}\right]}{K_{SUC}K_{Pi}}+\frac{\left[SUC_{m}\right]\left[Pi_{c}\right]}{K_{SUC}K_{Pi}} \end{matrix}\right)}$ (A42)

$J_{ DCC(SUC)}=\frac{T_{\max f}\left(\frac{\left[Pi_{m}\right]\left[SUC_{c}\right]}{K_{Pi}K_{SUC}}-\frac{\left[Pi_{c}\right]\left[SUC_{m}\right]}{K_{Pi}K_{SUC}}\right)}{\left(\begin{matrix} 1+\frac{\left[Pi_{m}\right]}{K_{Pi}}+\frac{\left[Pi_{c}\right]}{K_{Pi}}+\frac{\left[MAL_{m}\right]}{K_{MAL}}+\frac{\left[MAL_{c}\right]}{K_{MAL}}+\frac{\left[SUC_{m}\right]}{K_{SUC}}+\frac{\left[SUC_{c}\right]}{K_{SUC}}+ \\ \frac{\left[MAL_{c}\right]\left[Pi_{m}\right]}{K_{MAL}K_{Pi}}+\frac{\left[MAL_{m}\right]\left[Pi_{c}\right]}{K_{MAL}K_{Pi}}+\frac{\left[SUC_{c}\right]\left[Pi_{m}\right]}{K_{SUC}K_{Pi}}+\frac{\left[SUC_{m}\right]\left[Pi_{c}\right]}{K_{SUC}K_{Pi}} \end{matrix}\right)}$ (A43)

### *Transport 7: Tricarboxylate Carrier (TCC)*

TCC is non-electrogenic, only HCIT^2-^ and MAL^2-^ are accepted as transport species of TCC. One proton must bind to CIT for transport process to occur.

HCIT_c_ + MAL_m_ ⇌ HCIT_m_ + MAL_c_

The flux equation for this antiporter is:

$J_{TCC}=\frac{T_{ \max f}\left(\frac{\left[MAL_{c}\right]\left[H_{m}\right]\left[CIT_{m}\right]}{K_{CIT}K_{H}K_{MAL}}-\frac{\left[MAL_{m}\right]\left[H_{c}\right]\left[CIT_{c}\right]}{K_{CIT}K_{MAL}K_{H}}\right)}{\begin{matrix} 1+\frac{\left[CIT_{m}\right]\left[H_{m}\right]}{K_{CIT}K_{H}}+\frac{\left[CIT_{c}\right]\left[H_{c}\right]}{K_{CIT}K_{H}}+\frac{\left[MAL_{c}\right]}{K_{MAL}}+\frac{\left[MAL_{m}\right]}{K_{MAL}} \\ +\frac{\left[CIT_{m}\right]\left[H_{m}\right]\left[MAL_{c}\right]}{K_{CIT}K_{H}K_{MAL}}+\frac{\left[CIT_{c}\right]\left[H_{c}\right]\left[MAL_{m}\right]}{K_{CIT}K_{H}K_{MAL}} \end{matrix}}$ (A44)

***Table A36: TCC model kinetic parameters***

| **Parameter** | **Definition** | **Value** |
| --- | --- | --- |
| *K_CIT_* | CIT apparent Michaelis constant | $1\times10^{-3} M$ |
| *K_MAL_* | MAL apparent Michaelis constant | $0.25\times10^{-3} M$ |
| *K_H_* | Proton apparent Michaelis constant | $1\times10^{-7} M$ |

### *Transport 8: Pyruvate-Hydrogen Cotransporter (PYRH)*

PYR_c_ + H_c_^+^ ⇌ PYR_m_ + H_m_^+^

$J_{ PYRH}=\frac{T_{\max f}\left(\frac{[PYR_{c}][H_{c}^{+}]}{K_{PYR}K_{H}}-\frac{[PYR_{m}][H_{m}^{+}]}{K_{PYR}K_{H}}\right)}{1+\frac{[PYR_{c}]}{K_{PYR}}+\frac{[PYR_{m}]}{K_{PYR}}+\frac{[H_{c}^{+}]}{K_{H}}+\frac{[H_{m}^{+}]}{K_{H}}+\frac{[PYR_{c}]}{K_{PYR}}\frac{[H_{c}^{+}]}{K_{H}}+\frac{[PYR_{m}]}{K_{PYR}}\frac{[H_{m}^{+}]}{K_{H}}}$ (A45)

***Table A37****:* ***PYRH model kinetic parameters***

| **Parameters** | **Definition** | **Value** |
| --- | --- | --- |
| *K_PYR_* | PYR apparent Michaelis constant | $0.24\times10^{-3} M$ |
| *K_H_* | H^+^ apparent Michaelis constant | $1\times10^{-7} M$ |

### *Transport 9: Inorganic Phosphate Carrier (PIC)*

Pi_c_ + H_c_^+^ ⇌ Pi_m_ + H_m_^+^

$J_{ PIC}=\frac{T_{\max f}\left(\frac{[Pi_{c}][H_{c}^{+}]}{K_{Pi}K_{H}}-\frac{[Pi_{m}][H_{m}^{+}]}{K_{Pi}K_{H}}\right)}{1+\frac{[Pi_{c}]}{K_{Pi}}+\frac{[Pi_{m}]}{K_{Pi}}+\frac{[H_{c}^{+}]}{K_{H}}+\frac{[H_{m}^{+}]}{K_{H}}+\frac{[Pi_{c}]}{K_{Pi}}\frac{[H_{c}^{+}]}{K_{H}}+\frac{[Pi_{m}]}{K_{Pi}}\frac{[H_{m}^{+}]}{K_{H}}}$ (A46)

***Table A38: PIC model kinetic parameters***

| **Parameters** | **Definition** | **Value** |
| --- | --- | --- |
| *K_Pi_* | Pi apparent Michaelis constant | $9.4\times10^{-3} M$ |
| *K_H_* | H^+^ apparent Michaelis constant | $1\times10^{-7} M$ |

### *Transport 10: ATP-ADP Anti-Transporter (ANT):*

ADP_c_ + ATP_m_ ⇌ ADP_m_ + ATP_c_

Free ATP carries four negative charges while free ADP carries three negative charges. Therefore, the transport process catalyzed by ANT is affected by membrane potential. The flux equation was modified based on (Wu et al., 2007) to account for the effect of membrane potential. The transport flux ATP vs. ADP via ANT is given by:$J_{ ANT}=\frac{T_{\max f}\left\{\exp\left(\frac{\beta_{ANT}F\Delta\Psi_{m}}{RT}\right)\frac{\left[ADP_{c}\right]\left[ATP_{m}\right]}{K_{ADP}K_{ATP}}-\exp\left(\frac{(\beta_{ANT}-1)F\Delta\Psi_{m}}{RT}\right)\frac{\left[ADP_{m}\right]\left[ATP_{c}\right]}{K_{ADP}K_{ATP}}\right\}}{\left(1+\frac{\left[ADP_{m}\right]}{K_{ADP}}+\frac{\left[ATP_{m}\right]}{K_{ATP}}\exp\left(\frac{\beta_{ANT}F\Delta\Psi_{m}}{RT}\right)\right)\left(1+\frac{\left[ADP_{c}\right]}{K_{ADP}}+\frac{\left[ATP_{c}\right]}{K_{ATP}}\exp\left(\frac{(\beta_{ANT}-1)F\Delta\Psi_{m}}{RT}\right)\right)}$ (A47)

***Table A39: ANT model kinetic parameters***

| **Parameters** | **Definition** | **Value** | **Source** |
| --- | --- | --- | --- |
| *K_ADP_* | ADP apparent Michaelis constant | $10\times10^{-6} M$ | (Palmieri, 1994) |
| *K_ATP_* | ATP apparent Michaelis constant | $10\times10^{-6} M$ | (Palmieri, 1994) |
| *β_ANT_* | ANT free energy barrier | 0.6 | (Wu et al., 2007) |

### *Transport 11: Glutamate-Hydrogen Cotransporter (GLUH)*

GLU_c_ + H_c_^+^ ⇌ GLU_m_ + H_m_^+^

$J_{GLUH}=\frac{T_{\max f}\left(\frac{\left[GLU_{c}\right]\left[H_{c}\right]}{K_{GLU}K_{H}}-\frac{\left[GLU_{m}\right]\left[H_{m}\right]}{K_{GLU}K_{H}}\right)}{1+\frac{\left[GLU_{c}\right]}{K_{GLU}}+\frac{\left[GLU_{m}\right]}{K_{GLU}}+\frac{\left[H_{c}\right]}{K_{H}}+\frac{\left[H_{m}\right]}{K_{H}}+\frac{\left[GLU_{c}\right]\left[H_{c}\right]}{K_{GLU}K_{H}}+\frac{\left[GLU_{m}\right]\left[H_{m}\right]}{K_{GLU}K_{H}}}$ (A48)

***Table A40: GLUH model kinetic parameters***

| **Parameter** | **Definition** | **Value** |
| --- | --- | --- |
| *K_GLU_* | GLU apparent Michaelis constant | $0.5\times10^{-3} M$ |
| *K_H_* | Proton apparent Michaelis constant | $1\times10^{-7} M$ |

### *Transport 12: Malate-Aspartate Shuttle (MAS)*

Four reactions and two transport fluxes are involved in malate-aspartate shuttle (MAS), namely cytosolic MDH (MDH1), mitochondrial MDH (MDH2), cytosolic GOT and mitochondrial GOT, OME and GAE.

**MDH1:** MAL_m_ + NAD_m_ ⇌ OXA_m_ + NADH_m_ + H_m_; $K'_{eq,MDH1}=e^{-\text{Δ}_{r}G_{MDH}^{'0}/RT}10^{pH_{m}-7}$

**MDH2:** OXA_c_ + NADH_c_ + H_c_⇌ MAL_c_ + NAD_c_; $K'_{eq,MDH2}=e^{-\text{Δ}_{r}(-G_{MDH}^{'0})/RT}10^{7-pH_{c}}$

**GOT1:** ASP_m_ +AKG_m_ ⇌ GLU_m_ + OXA_m_; $K'_{eq,GOT1}=e^{-\text{Δ}_{r}G_{GOT}^{'0}/RT}$

**GOT2:** GLU_c_ + OXA_c_ ⇌ ASP_c_ +AKG_c_; $K'_{eq,GOT2}=e^{-\text{Δ}_{r}(-G_{GOT}^{'0})/RT}$

**OME:** AKG_m_ + MAL_c_ ⇌ AKG_c_ + MAL_m_; $K'_{eq,OME}=1$

**GAE:** ASP_c_ + HGLU_m_ ⇌ ASP_m_ + HGLU_c_; $K'_{eq,GAE}=\exp\left(\frac{F\Delta\Psi_{m}}{RT}\right)$

For simplicity and in order to reduce the number of unknown model parameters, the overall process is represented mathematically as one transporter by summing the above six processes,

NADH_c_ + NAD_m_ ⇌ NADH_m_ + NAD_c_

while accounting for the thermodynamic constraints of the four reactions and two transport processes. For instance, the transporter GAE is driven by ΔΨ_m_, and the equilibrium constants of MDH1 and MDH2 are affected by pH. Therefore, the apparent equilibrium constant for the malate-asparate shuttle in the model is

$K'_{eq,MA}=K'_{eq,GAE}K'_{eq,MDH1}K'_{eq,MDH2}K'_{eq,GOT1}K'_{eq,GOT2}K'_{eq,OME}=\exp\left(\frac{F\Delta\Psi_{m}}{RT}\right)\times10^{pH_{m}-pH_{c}}$, (A49)

and the overall flux equation is defined as

$J_{MAS}=\frac{\frac{T_{\max f}}{K_{NADH}K_{NAD}}\left(\left[NADH_{c}\right]\left[NAD_{m}\right]-\frac{\left[NADH_{m}\right]\left[NAD_{c}\right]}{K_{eq}^{'}}\right)}{\left(1+\frac{\left[NADH_{c}\right]\left[NAD_{m}\right]}{K_{NADH}K_{NAD}}+\frac{\left[NADH_{m}\right]\left[NAD_{c}\right]}{K_{NADH}K_{NAD}}\right)}$ (A50)

***Table A41: MAS model kinetic parameters***

| **Parameters** | **Definition** | **Value** |
| --- | --- | --- |
| *K_NADH_ K_NAD_* | Product of NADH apparent Michaelis constant and NAD apparent Michaelis constant | $1\times10^{-6} M$ |

### *Transport 13: Passive Proton Leak (Wu et al., 2007)*

$J_{HLEAK}=\frac{T_{\max f}}{K_{H}}\left(\left[H_{i}^{+}\right]\exp\left(\frac{F\Delta\Psi}{2RT}\right)-\left[H_{m}^{+}\right]\exp\left(-\frac{F\Delta\Psi}{2RT}\right)\right)$ (A51)

***Table A42: Proton leak model kinetic parameters***

| **Parameters** | **Definition** | **Value** |
| --- | --- | --- |
| *K_H_* | H^+^ apparent Michaelis constant | $1\times10^{-7} M$ |

## Part S4: Governing mass balance equations for the lung tissue bioenergetics model

### Reservoir region:

$V_{r}\frac{d[GLC_{r}]}{dt}=F\times\left([GLC_{b}]-[GLC_{r}]\right)$; (A52)

$V_{r}\frac{d[PYR_{r}]}{dt}=F\times\left([PYR_{b}]-[PYR_{r}]\right);$ (A53)

$V_{r}\frac{d[LAC_{r}]}{dt}=F\times\left([LAC_{b}]-[LAC_{r}]\right);$ (A54)

$V_{r}\frac{d[Pi_{r}]}{dt}=F\times\left([Pi_{b}]-[Pi_{r}]\right);$ (A55)

$V_{r}\frac{d[H_{r}^{+}]}{dt}=F\times\left([H_{b}^{+}]-[H_{r}^{+}]\right);$ (A56)

where F is the perfusion flow rate of isolated perfused rat lung system. Initial concentrations for the various species are dependent on the experimental conditions that are being simulated.

$V_{b}\frac{d[GLC_{b}]}{dt}=F\times\left([GLC_{r}]-[GLC_{b}]\right)-J_{GLUT};$ (A57)

$V_{b}\frac{d[PYR_{b}]}{dt}=F\times\left([PYR_{r}]-[PYR_{b}]\right)-J_{PYRT};$ (A58)

$V_{b}\frac{d[LAC_{b}]}{dt}=F\times\left([LAC_{r}]-[LAC_{b}]\right)-J_{LACT};$ (A59)

$V_{b}\frac{d[Pi_{b}]}{dt}=F\times\left([Pi_{r}]-[Pi_{b}]\right)-J_{PIT};$ (A60)

$V_{b}\frac{d[H_{b}^{+}]}{dt}=F\times\left([H_{r}^{+}]-[H_{b}^{+}]\right)-J_{LACT}-J_{PYRT};$ (A61)

### Initial concentrations for the various species are dependent on the experimental conditions that are being simulated.

### Cytosolic region

$V_{c}\frac{d[GLC_{c}]}{dt}=J_{GLUT}-J_{HK};$ $[GLC_{c}]\left(t=0\right)=0.56mM$ (A62)

$V_{c}\frac{d[G6P_{c}]}{dt}=J_{HK}-J_{PGI}-(1-\frac{5}{6})J_{PPP};$ $[G6P_{c}]\left(t=0\right)=0.278mM$ (A63)

$V_{c}\frac{d[F6P_{c}]}{dt}=J_{PGI}-J_{PFK};$ $[F6P_{c}]\left(t=0\right)=0.0486mM$ (A64)

$V_{c}\frac{d[F16BP_{c}]}{dt}=J_{PFK}-J_{ALD};$ $[F16BP_{c}]\left(t=0\right)=0.0667mM$ (A65)

$V_{c}\frac{d[GAP_{c}]}{dt}=2\times J_{ALD}-J_{GAPDH};$ $[GAP_{c}]\left(t=0\right)=0.0889mM$ (A66)

$V_{c}\frac{d[BPG_{c}]}{dt}=J_{GAPDH}-J_{PGK};$ $[BPG_{c}]\left(t=0\right)=0.0889mM$ (A67)

$V_{c}\frac{d[PEP_{c}]}{dt}=J_{PGK}-J_{PK};$ $[PEP_{c}]\left(t=0\right)=5.39\mu M$ (A68)

$V_{c}\frac{d[PYR_{c}]}{dt}=J_{PK}-J_{LDH}+J_{PYRT}-J_{PYRH};$ $[PYR_{c}]\left(t=0\right)=0.1mM$ (A69)

$V_{c}\frac{d[LAC_{c}]}{dt}=J_{LDH}+J_{LACT};$ $[LAC_{c}]\left(t=0\right)=1mM$ (A70)

$V_{c}\frac{d[PG6_{c}]}{dt}=0;$ $[PG6_{c}]\left(t=0\right)=0.3mM$ (A71)

$V_{c}\frac{d[MAL_{c}]}{dt}=-J_{DCC(MAL)}-J_{TCC};$ $[MAL_{c}]\left(t=0\right)=2mM$ (A72)

$V_{c}\frac{d[CIT_{c}]}{dt}=J_{TCC};$ $[CIT_{c}]\left(t=0\right)=2mM$ (A73)

$V_{c}\frac{d[SUC_{c}]}{dt}=-J_{DCC(SUC)};$ $[SUC_{c}]\left(t=0\right)=2mM$ (A74)

$V_{c}\frac{d[GLU_{c}]}{dt}=-J_{GLUH};$ $[GLU_{c}]\left(t=0\right)=6mM$ (A75)

$V_{c}\frac{d[ASP_{c}]}{dt}=0;$ $[ASP_{c}]\left(t=0\right)=6mM$ (A76)

$V_{c}\frac{d[Pi_{c}]}{dt}=J_{PIT}-J_{PIC}+J_{DCC(SUC)}+J_{DCC(MAL)}-J_{GAPDH}+J_{ATPase};$ $[Pi_{c}]\left(t=0\right)=2.5mM$ (A77)

$V_{c}\frac{d[AMP_{c}]}{dt}=-J_{AK};$ $[AMP_{c}]\left(t=0\right)=0.21mM$ (A78)

$V_{c}\frac{d[ADP_{c}]}{dt}=J_{HK}+J_{PFK}-J_{PGK}-J_{PK}+2\times J_{AK}+J_{ATPase}-J_{ANT};$ $[ADP_{c}]\left(t=0\right)=0.29mM$ (A79)

$V_{c}\frac{d[ATP_{c}]}{dt}=-J_{HK}-J_{PFK}+J_{PGK}+J_{PK}-J_{AK}-J_{ATPase}+J_{ANT};$ $[ATP_{c}]\left(t=0\right)=2.4mM$ (A80)

$V_{c}\frac{d[NADH_{c}]}{dt}=J_{GAPDH}-J_{LDH}-J_{MAS};$ $[NADH_{c}]\left(t=0\right)=0.68\mu M$ (A81)

$V_{c}\frac{d[NAD_{c}]}{dt}=-V_{c}\frac{d[NADH_{c}]}{dt};$ $[NAD_{c}]\left(t=0\right)=340\mu M$ (A82)

$V_{c}\frac{d[NADPH_{c}]}{dt}=2\times J_{PPP}-J_{GSR};$ $[NADPH_{c}]\left(t=0\right)=34.6\mu M$ (A83)

$V_{c}\frac{d[NADP_{c}]}{dt}=-V_{c}\frac{d[NADPH_{c}]}{dt};$ $[NADP_{c}]\left(t=0\right)=24.2\mu M$ (A84)

$V_{c}\frac{d[GSSG_{c}]}{dt}=-J_{GSR}+J_{GP};$ $[GSSG_{c}]\left(t=0\right)=0.21mM$ (A85)

$V_{c}\frac{d[GSH_{c}]}{dt}=2J_{GSR}-2J_{GP};$ $[GSH_{c}]\left(t=0\right)=4.1mM$ (A86)

$V_{c}\frac{d[H_{2}O_{2,c}]}{dt}=0;$ $[H_{2}O_{2,c}]\left(t=0\right)=0.03mM$ (A87)

$V_{c}\frac{d[H_{c}]}{dt}=0;$ $[H_{c}]\left(t=0\right)=0.794\times10^{-7}M$,(pH = 7.1) (A88)

### Mitochondria region

The ordinary differential equations in mitochondria are the same as in the mitochondria model, for completeness, the equations are listed as below

$V_{m}\frac{d[GLU_{m}]}{dt}=J_{GOT}+J_{GLUH};$ $[GLU_{c}]\left(t=0\right)=6mM$ (A89)

$V_{m}\frac{d[ASP_{m}]}{dt}=-J_{GOT};$ $[ASP_{m}]\left(t=0\right)=6mM$ (A90)

$V_{m}\frac{d[PYR_{m}]}{dt}=-J_{PDH}+J_{PYRH};$ $[PYR_{m}]\left(t=0\right)=0.025mM$ (A91)

$V_{m}\frac{d[OXA_{m}]}{dt}=J_{MDH}-J_{CITS}+J_{GOT};$ $[OXA_{m}]\left(t=0\right)=0.001mM$ (A92)

$V_{m}\frac{d[CIT_{m}]}{dt}=+J_{CITS}-J_{ICDH}-J_{TCC};$ $[CIT_{m}]\left(t=0\right)=3mM$ (A93)

$V_{m}\frac{d[AKG_{m}]}{dt}=J_{ICDH}-J_{AKGDH}-J_{GOT};$ $[AKG_{m}]\left(t=0\right)=3mM$ (A94)

$V_{m}\frac{d[SCA_{m}]}{dt}=J_{AKGDH}-J_{SCAS};$ $[SCA_{m}]\left(t=0\right)=92.5\mu M$ (A95)

$V_{m}\frac{d[SUC_{m}]}{dt}=J_{SCAS}-J_{S\text{DH}}+J_{DCC(SUC)};$ $[SUC_{m}]\left(t=0\right)=2mM$ (A96)

$V_{m}\frac{d[FUM_{m}]}{dt}=J_{S\text{DH}}-J_{FH};$ $[FUM_{m}]\left(t=0\right)=3mM$ (A97)

$V_{m}\frac{d[MAL_{m}]}{dt}=J_{FH}-J_{MDH}+J_{DCC(MAL)}+J_{TCC};$ $[MAL_{m}]\left(t=0\right)=3mM$ (A98)

$V_{m}\frac{d[NADH_{m}]}{dt}=J_{MAS}+J_{P\text{DH}}+J_{ICDH}+J_{AKGDH}+J_{MDH}-J_{CI};$ $[NADH_{m}]\left(t=0\right)=1.3mM$ (A99)

$V_{m}\frac{d[NAD_{m}^{+}]}{dt}=-V_{m}\frac{d[NADH_{m}]}{dt};$ $[NAD_{m}]\left(t=0\right)=0.433mM$ (A100)

$V_{m}\frac{d[ATP_{m}]}{dt}=-J_{ANT}+J_{NDK}+J_{CV};$ $[ATP_{m}]\left(t=0\right)=3.2mM$ (A101)

$V_{m}\frac{d[ADP_{m}]}{dt}=-V_{m}\frac{d[ATP_{m}]}{dt};$ $[ADP_{m}]\left(t=0\right)=3.2mM$ (A102)

$V_{m}\frac{d[GTP_{m}]}{dt}=J_{SCAS}-J_{NDK};$ $[GTP_{m}]\left(t=0\right)=3.2mM$ (A103)

$V_{m}\frac{d[GDP_{m}]}{dt}=-V_{m}\frac{d[GTP_{m}]}{dt};$ $[GDP_{m}]\left(t=0\right)=3.2mM$ (A104)

$V_{m}\frac{d[ACoA_{m}]}{dt}=J_{PDH}-J_{CITS};$ $[ACoA_{m}]\left(t=0\right)=0.37mM$ (A105)

$V_{m}\frac{d[CoA_{m}]}{dt}=-J_{P\text{DH}}+J_{CITS}+J_{SCAS}-J_{AKG\text{DH}};$ $[COA_{m}]\left(t=0\right)=0.46mM$ (A106)

$V_{m}\frac{d[Pi_{m}]}{dt}=J_{PIC}-J_{SCAS}-J_{CV}-J_{DCC(MAL)}-J_{DCC(SUC)};$ $[Pi_{m}]\left(t=0\right)=2mM$ (A107)

$V_{m}\frac{d[H_{m}^{+}]}{dt}=+\frac{2.303[H_{m}^{+}]}{\beta_{mit}}\left(\begin{matrix} J_{HLEAK}-J_{TCC}+2J_{PIC}+J_{GLUH}+(1-2)J_{PDH} \\ +2J_{CITS}+J_{AKGDH}+J_{SCAS}+J_{MDH}-\left(4+1\right)J_{CI} \\ -2J_{CIII}-4J_{CIV}+\left(3-1\right)J_{CV} \end{matrix}\right);$ $[H_{m}^{+}]\left(t=0\right)=0.251\times10^{-7}M$, (pH = 7.6) (A108)

$V_{m}\frac{d[UQ_{m}]}{dt}=-J_{CI}-J_{SDH}+J_{CIII};$ $[UQ_{m}]\left(t=0\right)=0.675mM$ (A109)

$V_{m}\frac{d[UQH_{2m}]}{dt}=-V_{m}\frac{d[UQ_{m}]}{dt};$ $[UQH_{2,m}]\left(t=0\right)=0.675mM$ (A110)

$\frac{d\Delta\Psi_{m}}{dt}=\frac{1}{C_{imm}}\left(\begin{matrix} 4J_{CI}+2J_{CIII}+4J_{CIV}-3J_{CV} \\ -J_{HLEAK}-J_{ANT}-J_{MAS} \end{matrix}\right);$ $[\Delta\Psi_{m}]\left(t=0\right)=130mV$ (A111)

**Oxygen consumption:**

Oxygen concentration was assumed constant in the isolated perfused rat lung experiments.

$\frac{d[O_{2}]}{dt}=0;$ $[O_{2}]\left(t=0\right)=0.296mM$ (A112)

**References:**

Abuelgassim, A.O., Salem, A.M., and Khoja, S.M. (1992). Allosteric control of 6-phosphofructo-1-kinase from rat lung. *Comp Biochem Physiol B* 101**,** 135-138.

Bakker, B.M., Michels, P.a.M., Opperdoes, F.R., and Westerhoff, H.V. (1997). Glycolysis in Bloodstream Form Trypanosoma brucei Can Be Understood in Terms of the Kinetics of the Glycolytic Enzymes. *Journal of Biological Chemistry* 272**,** 3207-3215.

Berg, J.M.T., J.L.; Stryer, L. (2002). "The Glycolytic Pathway Is Tightly Controlled.," in *Biochemistry*. 5th edition ed (New York: W H Freeman).

Bohme, C.C., Arscott, L.D., Becker, K., Schirmer, R.H., and Williams, C.H., Jr. (2000). Kinetic characterization of glutathione reductase from the malarial parasite Plasmodium falciparum. Comparison with the human enzyme. *J Biol Chem* 275**,** 37317-37323.

Campos, G., Guixe, V., and Babul, J. (1984). Kinetic mechanism of phosphofructokinase-2 from Escherichia coli. A mutant enzyme with a different mechanism. *J Biol Chem* 259**,** 6147-6152.

Carbonell, J., Marco, R., Felíu, J.E., and Sols, A. (1973). Pyruvate Kinase. *European Journal of Biochemistry* 37**,** 148-156.

Connolly, N.M., D'orsi, B., Monsefi, N., Huber, H.J., and Prehn, J.H. (2016). Computational Analysis of AMPK-Mediated Neuroprotection Suggests Acute Excitotoxic Bioenergetics and Glucose Dynamics Are Regulated by a Minimal Set of Critical Reactions. *PLoS One* 11**,** e0148326.

Cortassa, S., Aon, M.A., Marbán, E., Winslow, R.L., and O'rourke, B. (2003). An Integrated Model of Cardiac Mitochondrial Energy Metabolism and Calcium Dynamics. *Biophysical Journal* 84**,** 2734-2755.

Cortassa, S., Sollott, S.J., and Aon, M.A. (2017). Mitochondrial respiration and ROS emission during beta-oxidation in the heart: An experimental-computational study. *PLoS Comput Biol* 13**,** e1005588.

Dash, R.K., and Beard, D.A. (2008). Analysis of cardiac mitochondrial Na(+)–Ca(2+) exchanger kinetics with a biophysical model of mitochondrial Ca(2+) handing suggests a 3: 1 stoichiometry. *The Journal of Physiology* 586**,** 3267-3285.

Dash, R.K., Qi, F., and Beard, D.A. (2009). A Biophysically Based Mathematical Model for the Kinetics of Mitochondrial Calcium Uniporter. *Biophysical Journal* 96**,** 1318-1332.

Gaitonde, M.K., Murray, E., and Cunningham, V.J. (1989). Effect of 6-Phosphogluconate on Phosphoglucose Isomerase in Rat Brain In Vitro and In Vivo. *Journal of Neurochemistry* 52**,** 1348-1352.

Hanau, S., Montin, K., Cervellati, C., Magnani, M., and Dallocchio, F. (2010). 6-Phosphogluconate Dehydrogenase Mechanism: EVIDENCE FOR ALLOSTERIC MODULATION BY SUBSTRATE. *The Journal of Biological Chemistry* 285**,** 21366-21371.

Heesbeen, E.C., Rijksen, G., Batenburg, J.J., Van Golde, L.M., and Staal, G.E. (1989). Phosphofructokinase in alveolar type II cells isolated from fetal and adult rat lung. *Biochim Biophys Acta* 1002**,** 388-394.

Heyde, E., and Ainsworth, S. (1968). Kinetic studies on the mechanism of the malate dehydrogenase reaction. *J Biol Chem* 243**,** 2413-2423.

Johnson, M.L., Hussien, R., Horning, M.A., and Brooks, G.A. (2011). Transpulmonary pyruvate kinetics. *Am J Physiol Regul Integr Comp Physiol* 301**,** R769-774.

Kerr, J.S., Baker, N.J., Bassett, D.J., and Fisher, A.B. (1979). Effect of perfusate glucose concentration on rat lung glycolysis. *Am J Physiol* 236**,** E229-233.

Li, X., Wu, F., Qi, F., and Beard, D.A. (2011). A database of thermodynamic properties of the reactions of glycolysis, the tricarboxylic acid cycle, and the pentose phosphate pathway. *Database* 2011**,** bar005-bar005.

Li, Y., Dash, R.K., Kim, J., Saidel, G.M., and Cabrera, M.E. (2009). Role of NADH/NAD+ transport activity and glycogen store on skeletal muscle energy metabolism during exercise: in silico studies. *Am J Physiol Cell Physiol* 296**,** C25-46.

Li, Y., Lai, N., Kirwan, J.P., and Saidel, G.M. (2012). Computational Model of Cellular Metabolic Dynamics in Skeletal Muscle Fibers during Moderate Intensity Exercise. *Cellular and molecular bioengineering* 5**,** 92-112.

Little, C., Olinescu, R., Reid, K.G., and O'brien, P.J. (1970). Properties and regulation of glutathione peroxidase. *J Biol Chem* 245**,** 3632-3636.

Mavis, R.D., and Stellwagen, E. (1968). Purification and subunit structure of glutathione reductase from bakers' yeast. *J Biol Chem* 243**,** 809-814.

Midelfort, C.F., Gupta, R.K., and Rose, I.A. (1976). Fructose 1,6-bisphosphate: isomeric composition, kinetics, and substrate specificity for the aldolases. *Biochemistry* 15**,** 2178-2185.

Mountassif, D., Baibai, T., Fourrat, L., Moutaouakkil, A., Iddar, A., El Kebbaj, M.S., and Soukri, A. (2009). Immunoaffinity purification and characterization of glyceraldehyde-3-phosphate dehydrogenase from human erythrocytes. *Acta Biochim Biophys Sin (Shanghai)* 41**,** 399-406.

Mulquiney, P.J., and Kuchel, P.W. (1999). Model of 2,3-bisphosphoglycerate metabolism in the human erythrocyte based on detailed enzyme kinetic equations: equations and parameter refinement. *Biochem J* 342 Pt 3**,** 581-596.

Palmieri, F. (1994). Mitochondrial carrier proteins. *FEBS Letters* 346**,** 48-54.

Schering, B., Eigenbrodt, E., Linder, D., and Schoner, W. (1982). Purification and properties of pyruvate kinase type M2 from rat lung. *Biochim Biophys Acta* 717**,** 337-347.

Sharma, H.K., Prasanna, H.R., and Rothstein, M. (1980). Altered phosphoglycerate kinase in aging rats. *J Biol Chem* 255**,** 5043-5050.

Vinnakota, K., Kemp, M.L., and Kushmerick, M.J. (2006). Dynamics of Muscle Glycogenolysis Modeled with pH Time Course Computation and pH-Dependent Reaction Equilibria and Enzyme Kinetics. *Biophysical Journal* 91**,** 1264-1287.

Wang, Y., Wei, L., Wei, D., Li, X., Xu, L., and Wei, L. (2016). Enzymatic Kinetic Properties of the Lactate Dehydrogenase Isoenzyme C(4) of the Plateau Pika (Ochotona curzoniae). *International Journal of Molecular Sciences* 17**,** 39.

Wu, F., Yang, F., Vinnakota, K.C., and Beard, D.A. (2007). Computer modeling of mitochondrial tricarboxylic acid cycle, oxidative phosphorylation, metabolite transport, and electrophysiology. *J Biol Chem* 282**,** 24525-24537.

Zhang, X., Dash, R.K., Jacobs, E.R., Camara, A.K.S., Clough, A.V., and Audi, S.H. (2018). Integrated computational model of the bioenergetics of isolated lung mitochondria. *PLoS One* 13**,** e0197921.
